# Mycosporine-Like Amino Acids (MAAs): Biology, Chemistry and Identification Features

**DOI:** 10.3390/ph14010063

**Published:** 2021-01-14

**Authors:** Vanessa Geraldes, Ernani Pinto

**Affiliations:** 1 School of Pharmaceutical Sciences, University of São Paulo, Avenida Prof. Lineu Prestes, 580, Butantã, São Paulo-SP CEP 05508-000, Brazil; vanessa.geraldes@usp.br; 2 Centre for Nuclear Energy in Agriculture, University of São Paulo, Piracicaba, Piracicaba-SP CEP 13400-970, Brazil

**Keywords:** mycosporines, mycosporine-like amino acids (MAAs), mass spectrometry, database, photoprotective compounds, UV-absorbing compounds

## Abstract

Mycosporines and mycosporine-like amino acids are ultra-violet-absorbing compounds produced by several organisms such as lichens, fungi, algae and cyanobacteria, especially upon exposure to solar ultraviolet radiation. These compounds have photoprotective and antioxidant functions. Mycosporine-like amino acids have been used as a natural bioactive ingredient in cosmetic products. Several reviews have already been developed on these photoprotective compounds, but they focus on specific features. Herein, an extremely complete database on mycosporines and mycosporine-like amino acids, covering the whole class of these natural sunscreen compounds known to date, is presented. Currently, this database has 74 compounds and provides information about the chemistry, absorption maxima, protonated mass, fragments and molecular structure of these UV-absorbing compounds as well as their presence in organisms. This platform completes the previous reviews and is available online for free and in the public domain. This database is a useful tool for natural product data mining, dereplication studies, research working in the field of UV-absorbing compounds mycosporines and being integrated in mass spectrometry library software.

## 1. Ultraviolet Radiation and Natural UV-Absorbing Compounds

The solar radiation reaching Earth is composed of infrared radiation (>800 nm), visible (photosynthetically active radiation, PAR, 400-750 nm) and ultraviolet radiation (UVR, 200–400 nm). UVR is divided into ultraviolet A (UVA, 320–400 nm), ultraviolet B (UVB, 280–320 nm) and ultraviolet C (UVC, 200–280 nm). Very small proportions of UVR contribute to the total irradiation on the Earth’s surface: 0% of UVC (which is completely absorbed by the ozone layer), less than 1% of UVB and less than 7% of UVA. However, this part of the solar spectrum is highly energetic [1]. Photosynthetic organisms harness PAR to convert light into chemical energy. An obligate requirement for PAR results in prolonged exposure to UVR, which is detrimental for most sun-exposed organisms. Furthermore, due to the ozone depletion, the amount of UV reaching Earth tends to increase [2]. In order to circumvent the photodamage, several organisms have evolved biochemical and mechanical defenses [3]. Among these is the ability to synthesize UV-screening compounds such as phenylpropanoids and flavonoids (in higher plants), melanin (in animals), mycosporines, mycosporine-like amino acids (in cyanobacteria, fungi, algae and animals) and several other photoprotective compounds [4]. As well as providing protection against ambient UV radiation, these substances have other physiological roles. In previous studies, the characteristics of a wide diversity of UV-absorbing compounds are explored [4].

## 2. Mycosporine-Like Amino Acids

Mycosporines and mycosporine-like amino acids (MAAs) are a large family of natural UV-absorbing sunscreens [5,6,7,8,9], having evolved for protection against chronic UVR exposure in a wide variety of organisms such as cyanobacteria, microalgae, fungi, seaweeds, corals, lichens, as well as in freshwater and marine animals [5,8]. Their presence is evidence not only of their importance as natural UV-screening compounds but also of their early phylogenetic innovation [4]. MAAs were first reported in the 1960s [10,11,12]. Since then, many studies have been carried out and several MAAs have been identified as well as information on their structure, distribution, properties and functions.

### 2.1. Physico-Chemical Characteristics of MAAs

**Figure 1:**
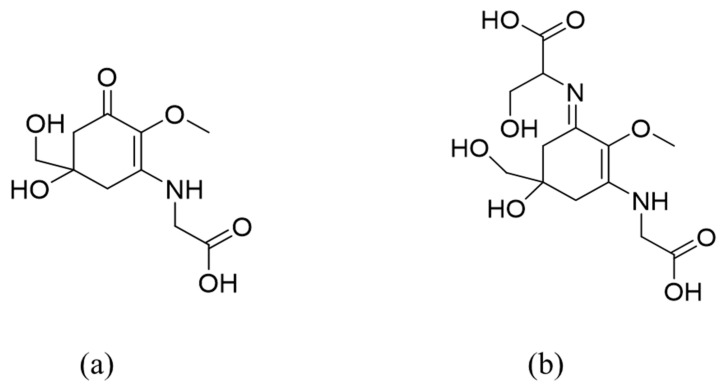
Examples of mycosporine-like amino-acids (MAAs) structures: (**a**) mycosporine-glycine (oxo-mycosporine); (**b**) shinorine (imino-mycosporine).

MAAs are low-molecular-weight (generally < 400 Da), colorless and water-soluble compounds. They are highly stable molecules under environmental conditions. They are composed of either an aminocyclohexenone or an aminocyclohexenimine ring, carrying nitrogen substituents. Aminocyclohexenone derivatives contain a cyclohexenone conjugated with an amino acid, such as mycosporine-glycine and mycosporine-taurine. Aminocyclohexenimine possesses a cyclohexenimine conjugated with a glycine or a methylamine attached to the third carbon atom and an amino acid or amino alcohol or enaminone chromophore to the first carbon atom (Figure 1) [13]. Glycosidic bonds or sulfate esters may occur within the imine group [14]. This group includes palythine, shinorine, porphyra-334, catenelline, hexose-bound porphyra-334, etc. MAA absorption maxima are between 268 and 362 nm, depending on their molecular structure, namely in the type of ring and substituents [15]. MAAs also have high molecular absorptivities (ε = 12.400–58.800 M^−1^·cm^−1^), and due to these characteristics, they are the strongest UVA-absorbing compounds in nature, and they are also effective against UVB radiations, which explains their potential role in photoprotection. MAAs are predominantly cytoplasmatic due to their high water solubility that enables MAAs to be easily dispersed in the cytoplasm [9]. Previous studies reviewed the physico-chemical properties of several MAAs [16].

### 2.2. Occurrence and Distribution in the Environment

Wittenberg et al. (1960) isolated, for the first time, compounds with high UV absorption from a siphonophore, *Physalia physalis* [10]. In 1965, mycosporines were discovered in fungal sporulating mycelia [12]. A few years later, MAAs have also been detected in corals and cyanobacteria from the Great Barrier Reef [11]. Since then, MAAs have been identified in a wide variety of organisms, such as heterotrophic bacteria [17], fungi [18], cyanobacteria [5,19], microalgae [20,21,22], macroalgae [23], lichens [6], invertebrates (e.g., dinoflagellates, sponges, corals, sea urchins and crustaceans) [2,24,25] and vertebrates (e.g., fishes) [26,27]. Several studies reported that animals can acquire MAAs from their food or through symbiosis and then subsequently accumulate them [28,29]. MAAs are not found in higher plants, in which UVR protection is provided by flavonoids, nor in higher vertebrates, in which the protective function is assumed by melanin [4]. MAAs are present especially in organisms that live in environments with high levels of UVR. Their composition varies according to the taxonomic group with the frequent coexistence of several MAAs with different absorption maxima allowing a more effective protective filter [8]. Several environmental factors, such as light, temperature, salinity and nutrients, influence the concentration of MAAs. Enhanced MAA contents, for example, were found in environments with a basic pH, a high ultraviolet radiation and high concentrations of phosphate and nitrate. Salinity, dissolved oxygen and variations of sea surface temperature also influence, in a secondary way, MAA content [30]. UV radiation is one of the most important factors that influences the accumulation of MAA and results in changes in the MAA profile of organisms. MAA synthesis is also affected by spectral variability and intensity [31,32]. Other factors, such as changes in salinity and nutrient availability, stimulate the production of MAAs. The content and composition of UV-absorbing MAAs are also affected by seasonal fluctuations [33,34]. These changes are mainly controlled by the solar radiation regime and nitrate regime. Guihéneuf et al. (2018) studies the temporal and spatial variability of mycosporine-like amino acids in seaweeds. An increase in total MAA contents in all species was induced by increasing daily light doses and irradiance levels, from winter to spring, but without clear significant correlations with light and/or temperature. Nutrient concentrations, in particular nitrate, appear to be a limiting factor for seaweed to accumulate MAAs when exposed to extreme light/irradiance stress [34].

### 2.3. MAA Biosynthesis

**Figure 2:**
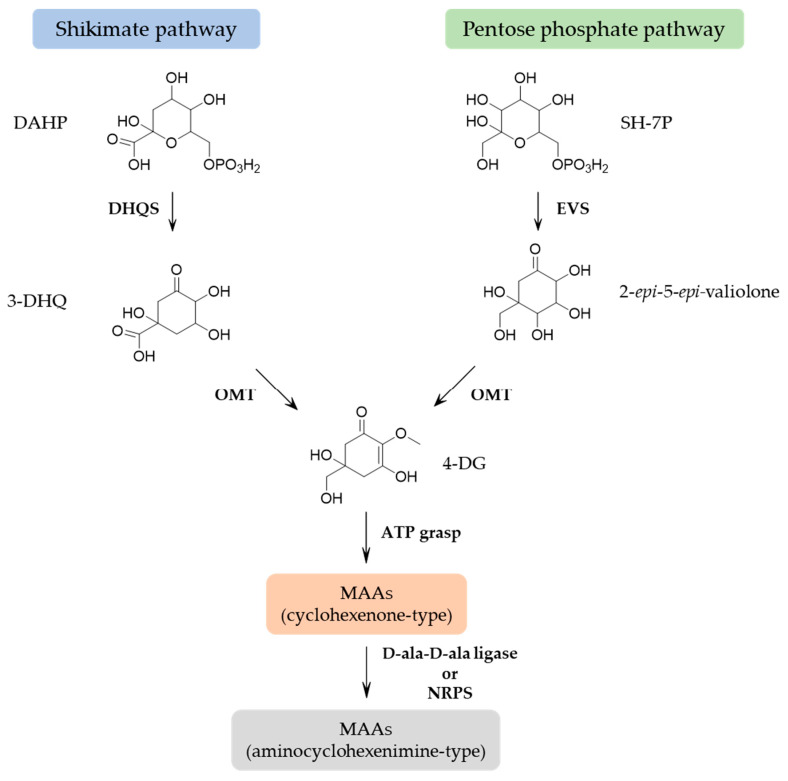
Proposed biosynthetic pathway of mycosporine-like amino acids. DAHP: 3-deoxy-D-arabino-heptulosonate phosphate, DHQS: 3-dehydroquinate synthase, 3-DHQ: 3-dehydroquinate, SH-7P: sedoheptulose-7-phosphate, EVS: cyclase-2-*epi*-5-*epi*-valiolone synthase, *O*MT: *O*-methyltransferase, 4DG: 4-deoxygadusol, ATP: adenosine triphosphate, NRPS: nonribosomal peptide synthase, D-ala-D-ala-ligase: D-alanyl-D-alanine ligase.

Cyanobacteria must have been the original MAA producer with the genes involved in MAA biosynthesis being transferred to other organisms, and MAAs may have been an early evolution to deal with cellular stress caused by UVR exposure [4]. Multiple lines of evidence support that MAAs are derived from conversion of the shikimate pathway [35], which is known for the synthesis of aromatic amino acids. The precursor of the six-membered carbon ring common to all MAAs is 3-dehydroquinate (3-DHQ). Three-DHQ transforms into gadusol and then 4-deoxygadusol (4-DG). However, contrasting evidence suggests that 4-DG is derived from conversion of the pentose phosphate pathway intermediate sedoheptulose-7-phosphate (SH-7P) [35,36,37]. Despite experimental data that support the pentose phosphate pathway, the use of the shikimate route inhibitors, such as glyphosate and tyrosine, has demonstrated the ability to abolish MAA biosynthesis in cyanobacteria [13] and corals [38]. Furthermore, the deletion of the gene encoding the enzyme cyclase-2-*epi*-5-*epi*-valiolone synthase (EVS) in cyanobacterium *Anabaena variabillis* ATCC 29413 still produced shinorine [39]. These results suggested that the shikimate pathway is the most predominant route for MAA synthesis in sufficient amounts to provide photoprotection, and the quantities of MAAs produced by the pentose phosphate pathway should have other biological functions [39]. However, there are clear links between the pentose phosphate and shikimate pathways. In both routes, 4-DG is the parent core structure of MAAs and the addition of glycine yielding mycosporine-glycine. This simple mono-substituted cyclohexenone-type MAA is a common intermediate in the production of di-substituted (aminocyclohexenimine-type) MAAs through the addition of a single amino acid residue (serine, threonine, etc.) yielding some common MAAs such as porphyra-334 and shinorine. This step encodes a nonribosomal peptide synthase (NRPS)-like protein or a D-alanyl-D-alanine ligase (D-ala-D-ala ligase) [13,36]. Figure 2 presents a proposed biosynthetic pathway of MAAs. Other MAAs are synthesized by modification in the attached side groups and nitrogen substituents (e.g., esterification, amidation, dehydration, decarboxylation, hydroxylation, sulfonation and glycosylation). The variations of amino acid side-chains are responsible for the difference in the absorption spectra of MAAs [15,40]. 

### 2.4. Heterologous Expression

The poor understanding of the biosynthesis pathways involved in the production of specific MAA is one of the reasons for the lack of widespread use of MAA in the industry in an economically viable way. Further understanding of these biosynthetic pathways can lead to easier large-scale production, for example, in a heterologous bacterial host [15,37]. Balskus et al. (2010) elucidated the biosynthesis of shinorine via heterologous expression in *Escherichia coli* [36]. The heterologous expression of MAAs in *Escherichia coli* also resulted in the production of 4-deoxygadusol, mycosporine-glycine, mycosporine-lysine and mycosporine-ornithine [36,37]. Shinorine and mycosporine-glycine-alanine have artificially been produced by heterologous expression in Actinomycetales [41]. Nowadays, most efforts are focused on the production of MAAs by genetically modified microorganisms as an alternative production of natural MAAs [9,37]. The heterologous expression of MAAs is significant from a biotechnological perspective, as MAAs are the active ingredient in next-generation sunscreens [37].

### 2.5. Chemical Synthesis and Analogs

The chemical synthesis of MAAs was motivated due to the low extraction yield from MAA-producing organisms and the need for large scale production. Several synthesized chemical structures were developed with interesting photoprotective and antioxidant properties [42,43,44,45]. The synthetic analogue of mycosporine-glycine, tetrahydropyridine, was considered hydrolytically and oxidatively stable for commercial application in sunscreens [46]. An efficient and environmentally friendly procedure by ultrasound and microwave for preparing MAA analogs was described by Andreguetti et al. (2013). These analogs showed high antioxidant effect [47]. Analogs with a high absorbance intensity in UVA and UVB regions, without in vitro cytotoxicity, were prepared by Nguyen et al. (2013) [48]. Recently, Losantos et al. (2017) developed a series of potential UV sunscreens with easy synthetic routes, providing a suitable source for their use in commercial products [42]. Bedoux et al. (2020) published a complete review of these chemical syntheses [49]. 

### 2.6. MAA Extraction, Identification and Quantification

#### 2.6.1. Extraction

Since MAAs are potential compounds to be used as sunscreens in cosmetic products, the extraction protocol must be optimized taking into account their physicochemical and absorption properties and must use environmentally friendly and inexpensive solvents [9,50]. Traditional extraction method is usually performed by a solid/liquid extraction from the raw material and is carried out on fresh or lyophilized samples with different temperature ranges [51,52]. Due to its high solubility in aqueous solution, extraction generally uses polar solvents, as aqueous ethanol or methanol. Chaves-Peña et al. (2020) compared the extraction in 20% aqueous methanol and in distilled water, and no significant differences were observed. According to their results, the drying and subsequent re-dissolution of the pellets declined total MAA concentrations [50]. Geraldes et al. (2020) developed a fast and efficient extraction protocol, without the need for pre-concentration procedures. This protocol uses only water and volatile additives as the extractor solvents, and the extracts were directly injected to a high-performance liquid chromatograph (HPLC). This extraction protocol is not only easy-to-handle but also uses solvents without certified toxic effects [32].

#### 2.6.2. Identification

HPLC was the most common method to separate and identify MAAs by using retention times and UV spectra. Although UV detection is sensitive because of high attenuation coefficients (ε) for MAAs, this method is poor in selectivity, since biosynthetic congeners can easily influence MAA identification [32]. Moreover, no commercial sources for standard compounds exist and few laboratories worldwide have the capacity to provide reference material against which structural elucidation of MAAs can be verified. In this sense, LC-MS is a good alternative to provide high sensitivity and selectivity for analysis of MAAs. A method for MAA identification was developed using an ultrahigh-performance liquid chromatography with diode array detection coupled to quadrupole time-of-flight mass spectrometry (UHPLC-DAD-QTOFMS) with an electrospray ionization source (ESI) and showed to be fast, reliable and a powerful tool for identification and screening of MAAs in several organisms, such as cyanobacteria, dinoflagellates, macroalgae and microalgae [42]. Regarding MAA purification, HPLC is the most used method. Geraldes et al. (2020) published a protocol using a semi-preparative HPLC-DAD fitted to a Luna C18 (2) column and 0.2% (v/v) formic acid solution as buffer A [32].

#### 2.6.3. Quantification

MAAs were often quantified based on molar attenuation coefficients using HPLC techniques. However, nowadays, liquid chromatography coupled with mass spectrometry (LC-MS) is the most common technique, because this method provides high sensitivity and selectivity for analysis of MAAs [32,51]. Whitehead and Hedges (2002) published a quantitative method that allowed the quantification of MAAs based on molecular weights, individual retention time and UV absorption maxima [53]. An LC-MS method using hydrophilic interaction chromatography (HILIC) was published by Hartmann et al. (2015) [54]. Although this method afforded good linear correlation coefficients, it did not account for some important validation parameters such as recovery and matrix effects. Geraldes et al. (2020) described a rapid quantitative method for LC-MS/MS analysis of MAAs that allowed the quantification of MAAs based on individual retention times, molecular weights and specific mass transitions using multiple reaction monitoring (MRM) experiments instead of a full scan [32]. This method has been thoroughly validated taking into account the ICH and EURACHEM guidelines for the following parameters: specificity, linearity, precision (repeatability and reproducibility within the laboratory), accuracy, extraction recovery, matrix effects and stability. In addition, the working range, as well as the limits of detection and quantification, were evaluated [32]. This technique improved the selectivity and sensitivity of the method, allowing mass distinction of isomeric compounds [16].

### 2.7. MAA Structural Elucidation

**Figure 3:**
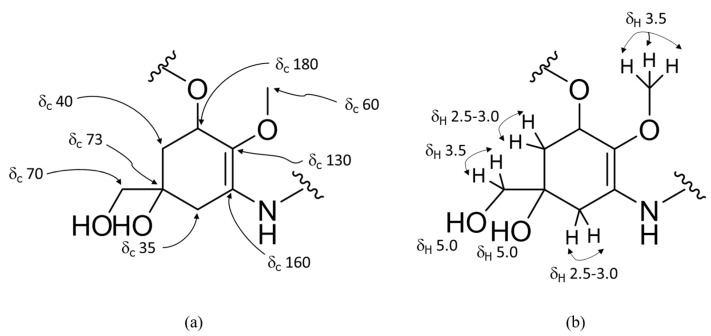
Structure of an oxomycosporine with NMR results for (**a**) ^13^C (125 MHz, D_2_O) and (**b**) ^1^H (500 MHz, D_2_O). For iminomycosporine ^13^C NMR (125 MHz, D_2_O) δ_C_ = 160 (C-1).

The structural elucidation of new derivatives of mycosporine-like amino acids is usually established by tandem mass spectrometry (MS/MS) and 1D and 2D nuclear magnetic resonance (NMR) spectroscopy [9,55]. Previous studies focusing on the fragmentation patterns of MAAs showed a loss of mass 15 when analyzed by positive mode ESI–MS/MS. This highly characteristic loss is due to elimination of a methyl radical CH_3_ [56,57]. The detection of the production [M + H − 59]^+^ related to the elimination of the methyl radical and subsequently CO_2_ is also common in MAAs [56]. Thus, the screening of these eliminations could be a suitable tool to assign the presence of novel MAAs in different samples by neutral loss or product ion scans in mass spectrometry analyzers. For aminocyclohexenimines, the elimination of the remaining lateral chain can produce the product ion *m/z* 186. This ion can undergo a fragmentation process to produce the ion *m/z* 155. Then, this product ion loses either NH_3_ or H_2_O by neutral elimination to produce the ions *m/z* 138 and 137, respectively [57]. These product ions could provide additional information concerning the occurrence of new MAAs in a crude extract. However, the presence of some product ions may be reduced or not occur in certain MAA fragmentation processes [58]. Thus, the selection of the product ions should be carried out carefully in analytical methods for the analysis of extracts containing potential novel MAAs. Regarding NMR spectroscopic data, they show characteristic patterns allowing them to be comparable. For example, oxo-mycosporines and imino-mycosporines can be distinguished through the chemical shift of C-1 in ^13^C NMR experiments, which is more around 180 ppm for oxo-mycosporines and 160 ppm for imino-mycosporines. The ^1^H NMR data usually revealed the presence of the coupling of the pairs of duplets from the diasterotopic methylenes, with a geminal coupling constant around 17 Hz, corresponding to the four protons on C-4 and C-6 that show chemical shifts around 30–40 ppm between them. The hydroxymethyl group at C-5 and the methoxyl group at C-2 usually appear very close around 3.60 ppm as two singlets [9,13,59] (Figure 3). 

### 2.8. MAA Photoprotective Role

The MAA photoprotective role against UVR is due to their absorption spectra and molar attenuation coefficients. Their absorption gradient (268–362 nm) cover most of the UVR spectrum (~295–400 nm) that reaches the Earth’s surface, while their high molar attenuation coefficients demonstrate how strongly MAAs absorb light at this wavelength range. The concentration of MAAs in organisms is directly related to their level of UVR exposure, which depends on latitude and altitude, seasonality and water depth [15,33,60,61]. They preferentially accumulate in tissues that receive the highest UVR exposure [62]. Photoprotection of MAAs has been demonstrated in a wide variety of species and prevents UVB-induced damage [63,64]. MAAs have been found in the cytoplasm of several cyanobacteria; however, in *Nostoc commune*, MAAs accumulate extracellularly, resulting in a more effective protection against ultraviolet radiation [65]. The presence of MAAs in animals supports their photoprotective role not only to producers but also to herbivores and even carnivores [8]. MAAs are also found in fossils that confirm their protective function against the UVR harmful effects in the early geological eras [66]. 

### 2.9. MAA Additional Protective Roles

Generally, MAA production is induced when organisms are exposed to UVR [67,68]. The photoprotective function is the most important role that MAAs play in nature. This is verified by the abundance of MAAs in organisms exposed to high UVR intensities [69]. However, MAAs can be produced constitutively in some species [5,13], and functionality could be linked to their individual structures [70]. Thus, MAAs may have additional protective roles in many other biological processes beyond their well-known UV sunscreen role. These compounds have convincingly demonstrated to possess physiologically relevant antioxidant properties [8,40]. Furthermore, MAAs are involved in osmotic regulation, desiccation and many other cellular functions [15,69]. An extensive review about these additional roles of MAAs has been carried out by Oren et al. (2007). However, the significance of these additional functions and the effects of different forms of stress on MAA synthesis are still poorly understood [69].

#### 2.9.1. Antioxidant and ROS Scavenging Function

UVR exposure generates oxidative stress and can produce reactive oxygen species (ROS) and DNA damage. This oxidative DNA damage can lead to mutations and inhibit DNA repair [71]. Antioxidants can reduce the harmful effects of ROS and can, thus, help to prevent oxidative stress [72]. Some MAAs may protect the cell not only by absorbing UVR and dissipating the energy as heat before it could reach the critical cellular targets but also as scavengers of free radicals due to their antioxidant role [31,73]. This function has been demonstrated in several MAAs from a wide variety of organisms [74,75,76,77,78,79]. MAAs have also been shown to prevent lipid peroxidation and superoxide radicals, blocking the aftereffect of oxidative damage [80]. Several assays demonstrated that MAAs have antioxidant properties and efficiently prevent oxidative stress through filtering and direct and indirect quenching mechanisms [81,82]. The antioxidant abilities of MAAs (ORAC values, lipid peroxidation inhibition, DPPH and ABTS radical extinction, singlet oxygen quenching, superoxide anion radical scavenging, hydrogen peroxide extinction activities and physiological activities) have been provided in recent reviews [40,83]. However, the exact mechanism is yet to be elucidated.

#### 2.9.2. Osmotic Stress

The concentrations of intracellular solute are directly related to the concentration of salt in which the cell lives [69]. Thus, osmotic stress is one of the stressors MAAs seem to have an action against. In hypersaline environments, cyanobacteria usually contain high concentrations of MAAs suggesting that these compounds may have an osmotic function helping the cells to cope with the high salinity. In these environments, cell dehydration and reactive oxygen species (ROS) production can occur, leading to oxidative stress. Through the synthesis of MAAs, osmotic balance can be restored [31]. Oren (1997) reported that a halotolerant cyanobacterium, inhabiting a gypsum crust, has an extremely high concentration of MAA (≥98 mM), being responsible for about 3% of the cells’ wet weight. It was observed that a reduction in the salt concentrations of its surroundings was accompanied with a rapid expulsion of MAAs [84]. Thus, MAAs may be involved in the adaptation of sea ice algae to osmotic variations. MAA production could be induced specifically either by exposure to UVB radiation or by osmotic stress, and a significant synergistic enhancement of MAA production was observed when both stress factors were combined. Although osmotic stress could induce MAA synthesis, MAAs play no significant role in attaining osmotic homeostasis [85]. Singh et al. (2008) proposed that salt treatment resulted in an increase in MAA content in the absence of UV radiation and had synergistic effects with UV stress [86]. Waditee-Sirisattha et al. (2013) recently demonstrated that the accumulation of MAAs, in a halotolerant cyanobacterium, was stimulated more under high salinity rather than under UVB radiation [87].

#### 2.9.3. Desiccation Stress

Desiccation is responsible for cell damage by affecting cytoplasmic components (such as DNA and proteins) and cell membrane fluidity. The production of polysaccharides and antioxidant compounds are some of the techniques to overcome the consequences of drying [64,88]. Few studies have been carried out to assess the effects on MAA concentration in microorganisms exposed to desiccation stress [63,64,89,90]. Desiccation stress leads to an increase in total MAA content. This group of compounds may be acting by modifying the structure of the extracellular matrix. Expulsion of the MAA is observed after rehydration. The combination of desiccation and irradiation stimulate MAA production. Colonies of fungi exposed to desiccation, UV radiation and nutrient scarcity contain high concentrations of mycosporine-glutaminol-glucoside. The survival and longevity potential of the vegetative hyphae of these fungi may be associated with the presence of these compounds [90]. Olsson-Francis et al. (2003) stated that cyanobacteria stressed experimentally by desiccation increased their MAA concentration. In this study, they suggest that the formation of an extracellular sheath may be related to desiccation [63]. Joshi et al. (2018) also proposed that desiccation together with UVB radiation led to an increase in the concentration of MAAs preventing and protecting cells from the harmful effects of these factors [64].

#### 2.9.4. Thermal Stress

There are a few reports of thermal stress protection by an increase in MAA induction in a range of organisms. Michalek-Wagner (2001) stated that MAA content was upregulated under heat stress, and their concentrations were further enhanced during simultaneous exposure to UV [91]. In contrast, some authors suggested that thermal stress had no effect on MAA production with or without UVR [85,86].

#### 2.9.5. Photosynthesis Accessory Pigments

An early study reported that MAAs may act as a photosynthetic accessory pigment due to its UVA absorption and subsequent production of small amounts of fluorescence at wavelengths close to the absorbance of chlorophyll-a. This result suggested that MAAs may increase photosynthetic efficiency. However, MAAs are only weakly fluorescent and are generally produced in environments of high irradiance, in which photosynthetic wavelengths are not the limiting factor for photosynthesis [92]. So far, this study has never been substantiated [69].

#### 2.9.6. Nitrogen Storage

MAAs are nitrogenous compounds that contain at least one nitrogen atom per molecule that can be released when required. Thus, MAAs may serve as an intracellular nitrogen storage [93]. A synergistic effect between ammonium ions and UVR was observed and resulted in an increase in MAA content [93]. If MAAs are accumulated as intracellular nitrogen storage compounds, nitrogen mobilization should occur whenever other suitable forms of nitrogen are absent. However, there is no evidence about the intracellular degradation of MAAs and the release of nitrogen atoms that support the proposal that MAAs may be nitrogen storage molecules [69]. 

#### 2.9.7. Reproductive Regulation

There is evidence that mycosporines and MAAs are involved in reproduction in fungi and marine invertebrates [69]. Mycosporines have been related to sporulating mycelia and were considered as biochemical markers for reproductive states of fungi or as reproduction markers [69,90]. Several studies reported that most MAAs reach their maximum concentration in ovaries and eggs at the time of ovarian reproductive maturity and spawning, which may be near the seasonal minima and maxima of solar irradiation [94,95]. Although a protective role is clearly demonstrated in marine invertebrate embryos, the exact function of MAAs in ovaries and eggs has not been determined [62]. 

#### 2.9.8. Ecological Interactions

Some MAAs play a role in ecological connectivity between organisms, as intraspecific alarm cues or cell-cell interaction tools, suggesting that MAAs can function as compounds of fundamental importance in marine ecosystems. The alarm cues are released in the ink secretion of sea hares and cause avoidance behaviors in neighboring conspecifics. The highest concentration of MAAs was concentrated in the defensive secretions and in the skin of these organisms [96]. In *M. aeruginosa* PCC 7806, shinorine was found to accumulate in an extracellular matrix, and this compound could be synthesized for its role in extracellular matrix formation and cell–cell interaction [97]. 

### 2.10. Cosmetical Application of MAAs as Sunscreen

As with other organisms, human exposure to ultraviolet radiation can cause damage, such as erythema or “sunburn” over the short term and premature skin aging and skin cancer over the long term [98]. Thus, protection against ultraviolet radiation is extremely important through the use of appropriate clothing and sunscreen, which are part of an overall prevention strategy [98,99]. Several photoprotective products are available on the market. They contain synthetic organic filters (e.g., oxybenzone, avobenzone, aminobenzoic acid), inorganic filters (e.g., titanium dioxide, zinc oxide) or a combination of both [100]. The frequent use of synthetic sunscreens can affect human health, causing allergic reactions, phototoxicity and endocrine disorders [101,102,103]. In addition, synthetic UVR filters are not environmentally friendly causing a negative impact in the marine life, including bioaccumulation in several species, hormonal changes and endocrine disruption in fish, hydrogen peroxide production and bleaching of corals [102,104,105,106]. This fact led Hawaii to ban some types of sunscreens, such as oxybenzone [107]. Subsequently, the Western Pacific Nation of Palau, the city of Key West, Florida and the US Virgin Islands have also passed similar bans [108]. Thus, there was a change in consumer trends with a strong demand in the cosmetics market for more natural products, since they are seen as safer and better and may be able to replace the existing ones. MAAs have been widely studied as natural alternatives to potentially toxic synthetic sunscreens and anti-aging products [8,15,83]. Several studies suggested that MAA have potential for the protection of human skin from a diverse range of adverse effects of solar UVR. Kageyama et al. (2019) provide an overview of MAAs, as potential anti-aging ingredients, which includes the molecular and cellular mechanisms through which MAAs might protect the skin. In addition to their UV-absorbing properties, these compounds have the potential to protect against skin aging, including antioxidative activity, anti-inflammatory activity, inhibition of protein-glycation and inhibition of collagenase activity [83]. MAAs are also highly stable over a wide range of temperature and pH [32,102]. These characteristics make them excellent cosmeceutical ingredients for skincare, cosmetics and pharmaceutical products [109,110]. However, only a few MAA products are currently available, and they still need to be exploited on a large scale. Mibelle AG Biochemistry developed a natural active compound, called Helioguard 365^®^, which contains MAA porphyra-334 and shinorine from the red seaweed *Porphyra umbilicalis*. Another MAA extract, named Helionori^®^, is also marketed by Gelyma [111]. Although these ingredients protect in UVA region, they provide minimal protection in the more damaging UVB range. Moreover, the MAA content in the formulation is usually very low when compared to the concentration of UVR filters in most sunscreen products. Thus, this ingredient ends up having a negligible influence on the SPF claims of the product [15]. Some MAA-based skin products have been marketed, such as Aethic Sôvée, a sunscreen with an environmentally friendly appeal. However, it is important to invest efforts to speed up the technological advancement of MAA application as an organic sunscreens [31].

### 2.11. Other Biotechnological Applications of MAAs 

**Figure 4:**
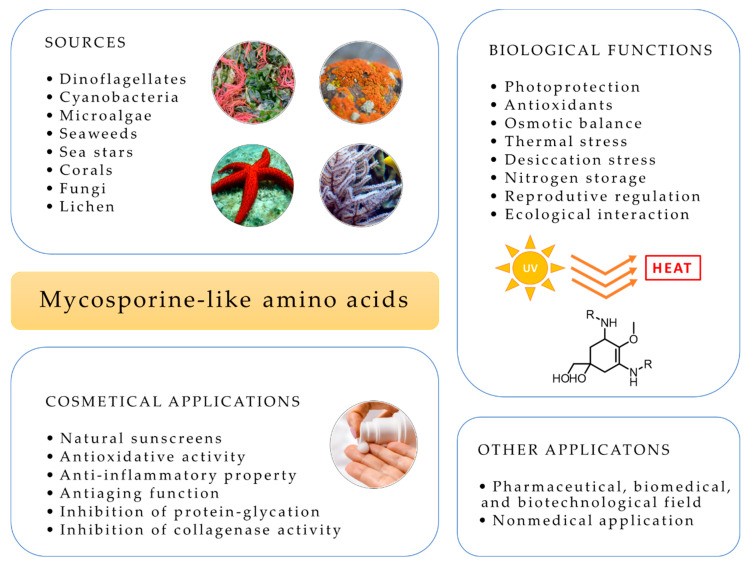
Mycosporine-like amino acids sources, biological functions and applications.

UV exposure alters the properties and durability of non-biological materials and affects their lifetime. MAAs have also nonmedical applications, as additives to protect plastics, paints and varnishes against UVR that exclusively consist of natural compounds [112]. These materials are biocompatible, photoresistant and thermoresistant and provide an efficient protection against UVA and UVB [112]. Figure 4 summarizes the main applications of MAAs including in cosmetics, biological functions and sources.

### 2.12. Patents on MAAs

Currently, there are already a large number of patents in international databases for several products and methods with MAAs. A list of patents on MAAs was obtained from patent databases such as the World Intellectual Property Organization, WIPO (http://www.wipo.int/patentscope/search/en/search.jsf), European Patent Office (http://www.epo.org/searching/free/espacenet.html) and United States Patents and Trademark Office (http://www.uspto.gov/patents/process/search/index.jsp). The search yielded a total of 48 patents on MAAs products and methods that are summarized in Supplementary Materials (Table S1).

### 2.13. MAA Database—MYCAS

There are several software for searching for commercial natural products. However, they do not cover most mycosporine-like amino acids, and they are not in the public domain. Over time, some MAA databases have been developed [22,23,40]. Although, none of them contains complete and detailed information on all mycosporines and mycosporine-like amino acids identified in the bibliography. Sinha et al. (2007) constructed an excellent database that provides information on various mycosporines and MAAs reported in several organisms. However, this database is now out of date [22]. Wada et al. (2015) published a study focused on MAAs with radical scavenging activities. This work resumed the structures and the physical and chemical properties of these MAAs. Nevertheless, this database did not include all MAAs known to date [40]. Sun et al. (2020) developed a database that summarized the studies related to MAAs in marine macroalgae. However, this work does not contain information on MAAs present in organisms other than macroalgae [23]. Thus, the purpose of this work is to cover this gap, so we present a database on mycosporines and mycosporine-like amino acids, called MYCAS. Our study covers the whole class of these natural sunscreen compounds known to date. This platform will be available online for free and will be in the public domain. It may also be incorporated into databases and search software of mass spectrometry for metabolomic and genomic studies. This platform provides information about their corresponding absorption maxima (λmax), molar attenuation (ε), molecular formula, exact mass, molecular structure, fragments and the organisms in which these compounds were found. Thus, MYCAS facilitate the search for mycosporine-like amino acids in different species and in dereplication studies. Our results are summarized in Supplementary Materials (Table S2). 

### 2.14. Main Tasks for Future Research and Perspectives

The optimization of MAA production on an industrial scale is essential to enable the use of these photoprotective compounds in cosmetical field. Thus, it is extremely important that resources are invested to optimize the production of MAAs and to increase the concentration in organisms, including GMO. At the same time, alternatives should be researched, such as heterologous expression or organic synthesis of analogues. The isolation and purification methods of MAAs are other processes that require further research. These techniques should be as simple and sustainable as possible to allow their application on a large scale. The commercial applicability of these compounds, as well as their impact on the environment and human health, should also be investigated to confirm the feasibility of using these photoprotective compounds on an industrial scale and warranty their safety.

## 3. Conclusions

Mycosporines and mycosporine-like amino acids are a large family of natural UV-absorbing compounds. To date, more than 70 molecules have been identified in several organisms from different phyla (Arthropoda, Cnidaria, Chordata, Cyanobacteria, Echinodermata, Fungi, Lichen, Macroalgae—Chlorophyta, Phaeophyta and Rhodophyta, Microalgae—Bacillariophyta, Charophyta, Chlorophyta, Dinoflagellata, Miozoa, Ochrophyta and Mollusca). Although there are several reviews on these photoprotective compounds, none include all of the MAAs known to date. In addition, the information is dispersed, and there is no review that summarizes all the information, namely the structural information, spectrometric data and presence in organisms. Here, we present an extremely complete database on mycosporines and mycosporine-like amino acids, called MYCAS, that covers the whole class of these natural sunscreen compounds. Currently, MYCAS has 74 compounds, with structural information and spectrometric data and will be updated annually. This platform is available online (http://www.cena.usp.br/ernani-pinto-mycas), for free and in the public domain. MYCAS allows users to access to all MAAs already described in nature. Our database may also be incorporated into in-house libraries, as well as mass spectrometry software for metabolomic and genomic studies. Thus, MYCAS is a useful tool for scientists working with MAAs and researchers in the field of developing UV-protecting cosmetics from natural sources. 

## Supplementary Materials

The following are available online at https://www.mdpi.com/1424-8247/14/1/63/s1, Table S1: Patents on mycosporines and mycosporine-like amino acids, Table S2: Mycosporines and mycosporine-like amino acids (MAAs) with their corresponding absorption maxima (λmax), molar absorptivities (ε), molecular formula, exact mass, molecular structure, fragments (underline font represents [M + H]^+^) and phylum.
