# Supplementary material for: Mycosporine-Like Amino Acids (MAAs): Biology, Chemistry and Identification Features
Source: Pharmaceuticals (Basel). 2021 Jan 14;14(1):63. doi: 10.3390/ph14010063 (PMC7828830; doi:10.3390/ph14010063)
Supplement: Supplementary file 1 [file pharmaceuticals-14-00063-s001.pdf]

Supplementary Materials

Table S1. Patents on mycosporines and mycosporine-like amino acids.

Year	Title	Patent n.º	Ref. [1]
1984	Mycosporine-like amino acid	JPS59137450	[1]
1987	Sunscreen compositions and compounds for use therein	WO1988002251<br>US5000946A	[2,3]
1990	UV-absorbing compounds and compositions	WO1990009995	[4]
2000	Solar radiation protection composition	WO2000024369<br>EP1123083<br>US6787147	[5–7]
2001	Algal extracts containing amino acid analogs of mycosporin are useful as dermatological protecting agents against ultraviolet radiation	FR2803201A1	[8]
2001	The utilization of natural pigments from lichens, cyanobacteria, fungi and plants for sun protection	WO2003020236<br>EP1424990A2<br>US20050129630<br>AU2002329025	[9–12]
2001	Topical cosmetic composition, useful for protecting skin and hair against sunlight, contains an extract from the red alga *Polysiphonia lanosa*	FR2803200	[13]
2002	Personal care compositions	WO2002039974<br>EP1341514A1	[14,15]
2002	Natural UV filters derived from pigments of lichens	IL0200725	[16]
2003	Extract having antioxidant activity and ultraviolet-absorbing activity, external preparation for skin, and food	JP2004238519A	[17]
2004	Cosmetic skin care products and cosmetic agents for protecting skin against premature aging	EP1473028	[18]
2004	Beta-glucuronidase inhibitors for use in deodorants and antiperspirants	US20040234466	[19]
2005	Amino-benzophenone UV filter formulations for the prevention of tanning	GB2412866	[20]
2005	Use of a mycosporin-type amino acid (porphyra 334) as an antioxidant	WO2007026035<br>ES2301293	[21,22]
2005	Use of a mycosporin-type amino acid (shinorine) as an antioxidant	WO2007026038<br>ES2301426	[23,24]
2005	Use of a mixture of mycosporin-type amino acids (asterin 330 + palythine) as an antioxidant	WO2007026037	[25]
2006	Use of a mycosporin-type amino acid (M-gly) as an antioxidant	WO2007026036	[26]
2006	Compositions comprising Porphyra and methods of making and using thereof	WO2007144779<br>EP2001311<br>US20070220806	[27–29]
2006	Sunscreen composition with extract of algae and lichens	ES2317741	[30]
2007	Extracts of Aphanizomenon flos-aquae and nutritional, cosmetic and pharmaceutical composition containing the same	WO2008000431<br>EP2032122<br>US20100021493	[31–33]
2007	Alphanizomenon flos aquae preparation, extracts and purified components thereof for the treatment of neurological, neurodegenerative and mood disorders	WO2008000430<br>EP2046354<br>US20090311286	[34–36]
2007	Mycosporin-like amino acid derivative having glycosyl group and method for producing the same	JP2009120562	[37]

Year	Title	Patent n.º	Ref. [1]
2007	Cosmetic including natural substance having sun-screening function	CN101061995	[38]
2008	Antioxidant compound, antioxidant algae extract and method for producing the same	JP2008247901A	[39]
2009	Cosmetic sunscreen composition	GB2472021	[40]
2010	Method for preparing UV screening nontoxic extract from red algae, and nontoxic sunscreen using same	WO2011096628<br>KR100969325<br>CN102740869<br>JP2013518871A	[41]
2011	Topical composition	WO2011158041	[42]
2012	Preparation method for laver mycosporine like amino acids porphyra-334	CN102659621	[43]
2012	Beauty product containing desert algae radiation-proof ingredient and natural medical whitening ingredient and preparation method thereof	CN102764206	[44]
2013	Imino compounds as protecting agents against ultraviolet radiations	WO2013181741<br>US20150152046	[45,46]
2013	Mycosporine-like amino acid and method for producing the same, UV protection agent, and antioxidant	JP6049200B2	[47]
2013	Mycosporin-like amino acids, production method thereof, UV protecting agents and antioxidants	JP2014227339	[48]
2013	Method for extracting mycosporine-like amino acid shinorine from microcystis	CN103755589	[49]
2013	Topical composition comprising transformed bacteria expressing a compound of interest	WO2014025938<br>US20140044677<br>US20160000701	[50,51]
2013	Synthesis of UV absorbing compounds	WO2014082124<br>US20150299124	[52,53]
2013	Anti-UV Composition for Skin External Application Comprising Peptide derivatives from Extract of Microalgae Comprising the Same	KR20150008238A	[54]
2014	UV absorbing compounds, compositions comprising same and uses thereof	WO2015006803<br>US20160244409	[55,56]
2015	Topical formulations for UV protection	WO2015195546	[57]
2015	Method for producing mycosporine-like amino acid using microbes	WO2015174427<br>US2017202762A1<br>EP3144392A1	[58–60]
2016	Novel mycosporine-like amino acid	WO2017082144A1	[61]
2016	Wound healing composition for skin external application comprising Mycosporine-like amino acid and Method for Preparing the Same	KR20170090690A	[62]
2016	Culture method capable of improving mycosporine-like amino acid content in umbilical laver	CN105684880B	[63]
2016	Extraction of mycosporine-like amino acids consisting of amino acid derivatives, separation and purification with chemically modified surface-modified activated carbon filler, and automation of their production	JP6058853B1	[64]
2017	Microorganism for producing a mycosporine-like amino acid method for producing a mycosporine-like amino acid using the same	CA3072748A1	[65]

Year	Title	Patent n.º	Ref. [1]
2018	A microorganism for producing a Mycosporine-like amino acid and a method for preparing a Mycosporine-like amino acid using the same	KR102003911B1	[66]
2018	Production of mycosporine-like amino acids in cyanobacteria	WO2019094447A2	[67]
2020	Microorganism for producing a mycosporine-like amino acid and method for producing a mycosporine-like amino acid using the same	US20200283810A1	[68]
2020	Solution containing stabilized mycosporine-like amino acid and method for producing the same	JP2020114871A	[69]

[1] Ref: References.

Table S2. Mycosporines and mycosporine-like amino acids (MAAs) with their corresponding absorption maxima (λmax), molar absorptivities (ε), molecular formula, exact mass, molecular structure, fragments (underline font represents $[M+H]^+$) and phylum, (-) no information available.

**Compound**<br>**Pubchem CID**	**λmax (nm)**<br>**ε ($M^{-1}.cm^{-1}$)**	**Molecular Formula**<br>**Exact mass**	**Molecular structure**<br>**Smiles**	**Fragments**	**Phylum**	**Ref.**
			**Mycosporine-like amino-acids precursors**			
4-Deoxygadusol<br>(Cyclohexenone)<br>85926716	269<br>15,700	$C_8H_{12}O_5$<br>188.07	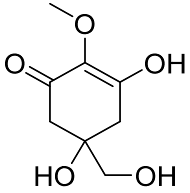<br>OC(CC(CO)(O)C1)=C(OC)C1=O	66.9, 70.0, 85.3, 95.0, 113.4, 160.0, 175.2, 189.2	Cnidaria, Equinodermata, Lichen, Chordata	[70–73]
Gadusol<br>195955	268<br>12,400	$C_8H_{12}O_6$<br>204.06	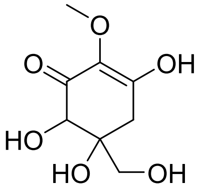<br>OC(CC(CO)(O)C1O)=C(OC)C1=O	58.3, 84.8, 117.5, 145.2, 160.0, 175.1, 203.7, 204.9, 205.4	Arthropoda, Chordata, Lichen	[70,72,74–76]
			**Oxo-mycosporines**			

Compound<br>Pubchem CID	λmax (nm)<br>ε ($M^{-1}.cm^{-1}$)	Molecular Formula<br>Exact mass	Molecular structure<br>Smiles	Fragments	Phylum	Ref.
Mycosporine-ethanolamine	310	$C_{10}H_{17}NO_5$<br>231.11	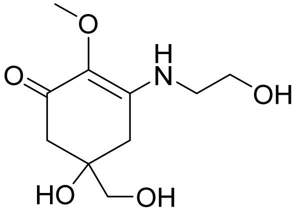<br>O=C1CC(CO)(O)CC(NCCO)=C1OC	-	Porifera	[77]
Mycosporine-glycine<br>14444486	310<br>28,100	$C_{10}H_{15}NO_6$<br>245.09	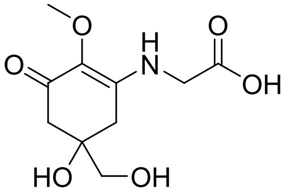<br>O=C1CC(CO)(O)CC(NCC(O)=O)=C1OC	57.1, 84.9, 100.1, 117.7, 143.9, 171.2, 231.7, 246.4	Arthropoda, Chlorophyta, Chordata, Cnidaria, Cyanobacteria, Dinoflagellata, Echinodermata, Lichen, Miozoa, Mollusca, Ochrophyta, Phaeophyta, Porifera, Rhodophyta	[70,71,78–83]
Mycosporine-alanine	310<br>640	$C_{11}H_{17}NO_6$<br>259.11	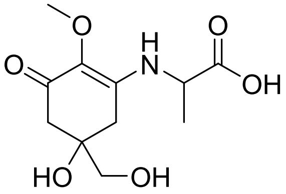<br>O=C1CC(CO)(O)CC(NC(C)C(O)=O)=C1OC	147, **171**, 42, 260,1	Fungi	[84]

Compound<br>Pubchem CID	λmax (nm)<br>ε ($M^{-1}.cm^{-1}$)	Molecular Formula<br>Exact mass	Molecular structure<br>Smiles	Fragments	Phylum	Ref.
Mycosporine-β-alanine	-<br>-	$C_{11}H_{17}NO_6$<br>259.11	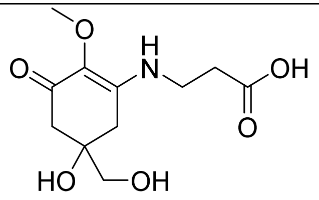<br>O=C1CC(CO)(O)CC(NCCC(O)=O)=C1OC	260,1	Cnidaria	[85,86]
Mycosporine-serinol<br>442866	310<br>27,270	$C_{11}H_{19}NO_6$<br>261.12	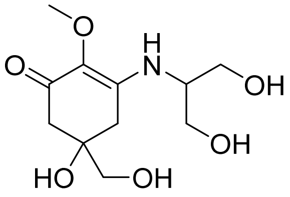<br>O=C1CC(CO)(O)CC(NC(CO)CO)=C1OC	184, 194, 212.1, 216, 243.1, 262.1	Fungi, Cyanobacteria	[87,88]
Mycosporine-GABA	310<br>28,900	$C_{12}H_{20}NO_6$<br>273.12	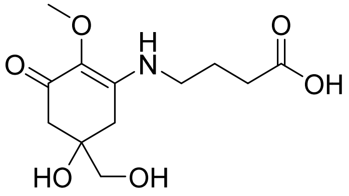<br>O=C1CC(CO)(O)CC(NCCCC(O)=O)=C1OC	87.1, 137.1, 186.2, 274.1	Cyanobacteria, Dinoflagellata	[81,89,90]

Compound<br>Pubchem CID	λmax (nm)<br>ε ($M^{-1}.cm^{-1}$)	Molecular Formula<br>Exact mass	Molecular structure<br>Smiles	Fragments	Phylum	Ref.
Mycosporine-serine	310<br>-	$C_{11}H_{17}NO_7$<br>275.10	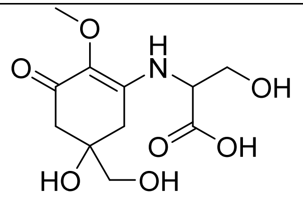<br>O=C1CC(CO)(O)CC(NC(C(O)=O)CO)=<br>C1OC	228, 236, 258,<br>276.1	Fungi	[91,92]
Mycosporine-2<br>101324823	310<br>-	$C_{13}H_{19}NO_6$<br>285.12	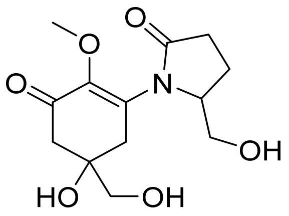<br>O=C1CC(CO)(O)CC(N2C(CO)CCC2=<br>O)=C1OC	286.1	Fungi	[93,94]
Mycosporine-taurine	309<br>28,100	$C_{10}H_{17}NO_7S$<br>295.07	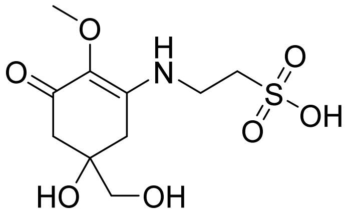<br>O=C1CC(O)(CO)CC(NCCS(=O)(O)=O)<br>=C1OC	57.1, 186.2, 236.1,<br>263.3, 277.2, 296.2	Cnidaria,<br>Cyanobacteria, Lichen	[70,95–97]

Compound<br>Pubchem CID	λmax (nm)<br>ε (M^{-1}.cm^{-1})	Molecular Formula<br>Exact mass	Molecular structure<br>Smiles	Fragments	Phylum	Ref.
Normycosporine-glutamine	320<br>-	$C_{12}H_{18}N_2O_7$<br>302.11	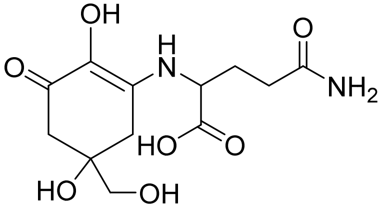<br>OC1=C(NC(C(O)=O)CCC(N)=O)CC(CO)(O)CC1=O	303.1	Fungi	[92,98]
Mycosporine-ornithine	310<br>-	$C_{13}H_{22}N_2O_6$<br>302.15	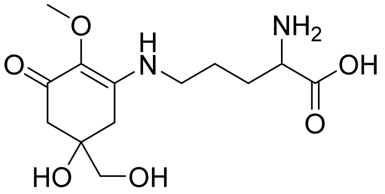<br>O=C1CC(O)(CO)CC(NCCCC(C(O)=O)N)=C1OC	191.1, 235.2, 267.0, 303.2	Cyanobacteria	[81,99]
Mycosporine-glutaminol<br>101835613	310<br>12,542	$C_{13}H_{22}N_2O_6$<br>302.15	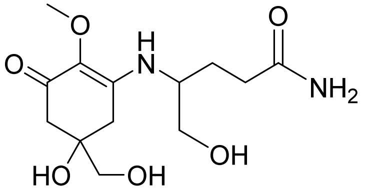<br>O=C1CC(O)(CO)CC(NC(CO)CCC(N)=O)=C1OC	235, 267, 285, 303.2	Cyanobacteria, Fungi, Lichen	[100–102]

Compound<br>Pubchem CID	λmax (nm)<br>ε ($M^{-1}.cm^{-1}$)	Molecular Formula<br>Exact mass	Molecular structure<br>Smiles	Fragments	Phylum	Ref.
Normycosporine-glutamic acid (artifact)	320<br>-	$C_{12}H_{17}NO_8$<br>303.10	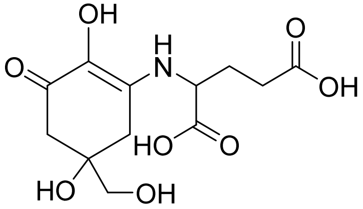<br>OC1=C(NC(C(O)=O)CCC(O)=O)CC(CO)(O)CC1=O	304.1	Fungi	[98]
Mycosporine-glutamicol<br>9882880	310<br>17,248	$C_{13}H_{21}NO_7$<br>303.13	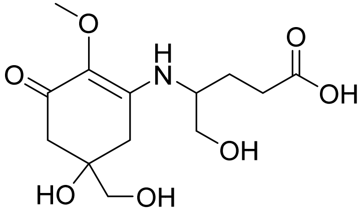<br>O=C1CC(CO)(O)CC(NC(CO)CCC(O)=O)=C1OC	178, 210, 236, 258, 268, 286, 304.1	Fungi, Lichen	[94,98,102,103]
Mycosporine-glutamine<br>101835612	310<br>-	$C_{13}H_{20}N_2O_7$<br>316.13	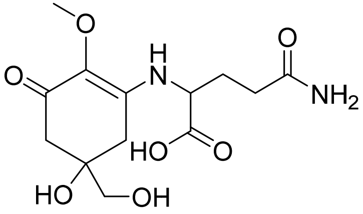<br>O=C1CC(CO)(O)CC(NC(C(O)=O)CCC(N)=O)=C1OC	317.1	Fungi	[98]

Compound<br>Pubchem CID	λmax (nm)<br>ε (M^{-1}.cm^{-1})	Molecular Formula<br>Exact mass	Molecular structure<br>Smiles	Fragments	Phylum	Ref.
Mycosporine-lysine	310<br>-	$C_{14}H_{24}N_2O_6$<br>316.16	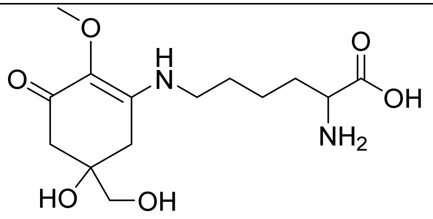<br>O=C1CC(CO)(O)CC(NCCCCC(N)C(O)=O)=C1OC	281.1, 317.2	Cyanobacteria	[99,104]
Mycosporine-glutamic acid<br>102095611	311<br>20,900	$C_{13}H_{19}NO_8$<br>317.11	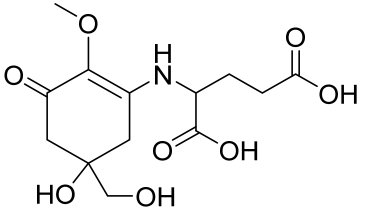<br>O=C1CC(CO)(O)CC(NC(C(O)=O)CCC(O)=O)=C1OC	225, 281, 299, 318.1	Fungi	[78,98,105]
Mycosporine-hydroxyglutamicol<br>101805382	310<br>-	$C_{13}H_{21}NO_8$<br>319.13	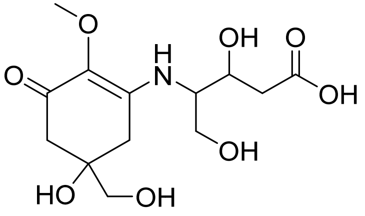<br>O=C1CC(CO)(O)CC(NC(CO)C(O)CC(O)=O)=C1OC	320.1	Lichen	[103]

Compound<br>Pubchem CID	λmax (nm)<br>ε ($M^{-1}.cm^{-1}$)	Molecular Formula<br>Exact mass	Molecular structure<br>Smiles	Fragments	Phylum	Ref.
Prasiolin<br>132581102	324<br>12,393	$C_{13}H_{19}NO_9$<br>333.11	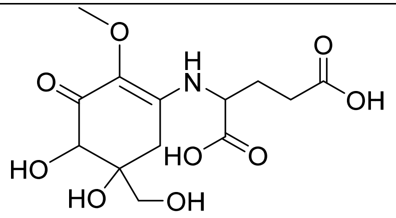<br>O=C1C(O)C(CO)(O)CC(NC(CCC(O)=O)C(O)=O)=C1OC	334.1	Chlorophyta	[106]
			**Imino-mycosporines**			
Palythine<br>16047608	320<br>35,500-36,200	$C_{10}H_{16}N_2O_5$<br>244.11	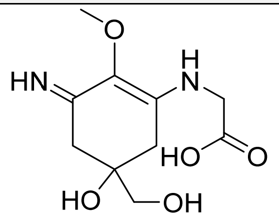<br>N=C1CC(CO)(O)CC(NCC(O)=O)=C1OC	137.2, 168.2, 185.9, 198.9, 209.2, 230.2, 245.1	Arthropoda, Bacillariophyta, Chlorophyta, Chordata, Cnidaria, Dinoflagellata, Echinodermata, Lichen, Miozoa, Mollusca, Ochrophyta, Phaeophyta, Porifera, Rhodophyta	[80–83,107–110]
N-methylpalythine = Aplysiapalythine C<br>-	330<br>-	$C_{11}H_{18}N_2O_5$<br>258.12	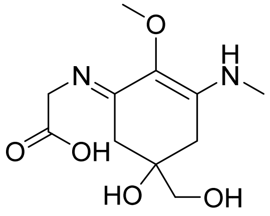<br>CNC1=C(OC)/C(CC(CO)(O)C1)=N/CC(O)=O	259.1	Cyanobacteria, Mollusca	[81,111]

Compound<br>Pubchem CID	λmax (nm)<br>ε (M-1.cm-1)	Molecular Formula<br>Exact mass	Molecular structure<br>Smiles	Fragments	Phylum	Ref.
Aplysiapalythine D	330<br>-	$C_{11}H_{18}N_2O_5$<br>258.12	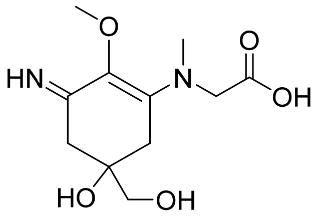<br>N=C1CC(CO)(O)CC(N(C)CC(O)=O)=C 1OC	259.1	Cyanobacteria	[112]
Dehydroxyl-usujirene	356<br>-	$C_{13}H_{19}N_2O_4$<br>267.13	(a)	209, 224, 268.2	Cyanobacteria	[96]
N-ethylpalythine<br>= Aplysiapalythine B	332<br>-	$C_{12}H_{20}N_2O_5$<br>272.14	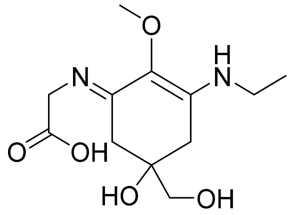<br>OC(CC(NCC)=C/1OC)(CO)CC1=N/CC (O)=O	273.1	Mollusca, Rhodophyta	[80,111,113]

Compound<br>Pubchem CID	λmax (nm)<br>ε ($M^{-1}.cm^{-1}$)	Molecular Formula<br>Exact mass	Molecular structure<br>Smiles	Fragments	Phylum	Ref.
Palythine-serine	320<br>10,500	$C_{11}H_{18}N_2O_6$<br>274.12	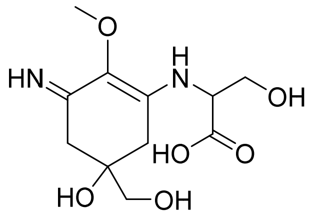<br>N=C1CC(CO)(O)CC(NC(CO)C(O)=O)=C1OC	260, 275.1	Cnidaria, Cyanobacteria	[81,114]
Usujirene (cis isomer)<br>15847474	357<br>-	$C_{13}H_{20}N_2O_5$<br>284.14	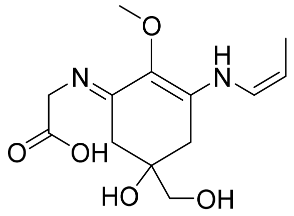<br>OC(CC(N/C=C\C)=C/1OC)(CO)CC1=N/CC(O)=O	197, 226, 241, 270, 285.1	Rhodophyta	[80,115,116]

Compound<br>Pubchem CID	λmax (nm)<br>ε ($M^{-1}.cm^{-1}$)	Molecular Formula<br>Exact mass	Molecular structure<br>Smiles	Fragments	Phylum	Ref.
Palythene<br>(trans isomer)<br>21773785	360<br>50,000	$C_{13}H_{20}N_2O_5$<br>284.14	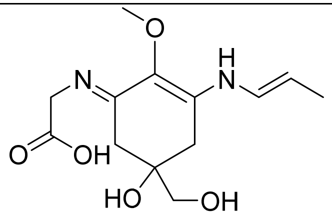<br>OC(CC(N/C=C/C)=C/1OC)(CO)CC1=N<br>/CC(O)=O	139.1, 185.0, 197.3, 205.0, 223.0, 241.2, 285.0	Arthropoda, Chlorophyta, Cnidaria, Lichen, Mollusca, Phaeophyta, Porifera, Rhodophyta	[70,80,83,110,117]
Mycosporine methylamine serine = N-methylmycosporine-serine	325<br>16,600	$C_{12}H_{20}N_2O_6$<br>288.13	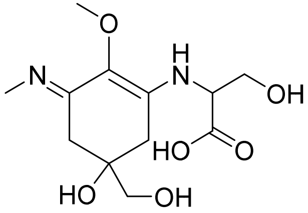<br>OC(CC(NC(CO)C(O)=O)=C/1OC)(CO)<br>CC1=N/C	274, 289.1	Cnidaria	[114]
Palythine–threonine	320<br>-	$C_{12}H_{20}N_2O_6$<br>288.13	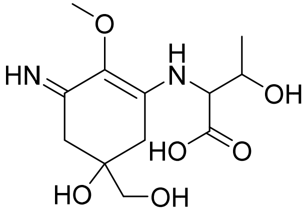<br>N=C1CC(CO)(O)CC(NC(C(C)O)C(O)=<br>O)=C1OC	169, 172, 230, 245, 256, 274, 289.1	Cnidaria, Cyanobacteria	[81,118,119]

Compound<br>Pubchem CID	λmax (nm)<br>ε ($M^{-1}.cm^{-1}$)	Molecular Formula<br>Exact mass	Molecular structure<br>Smiles	Fragments	Phylum	Ref.
Asterina-330<br>13194807	330<br>43,800	$C_{12}H_{20}N_2O_6$<br>288.13	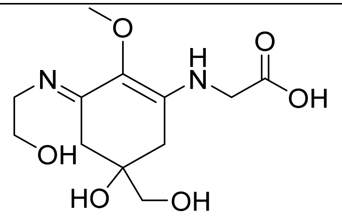<br>OC(CC(NCC(O)=O)=C/1OC)(CO)CC1=N/CCO	137, 167.8, 186.0, 198.4, 230.2, 243.1, 273.1, <u>289.2</u>	Arthropoda, Chlorophyta, Chordata, Echinodermata, Lichen, Mollusca, Ochrophyta, Phaeophyta, Rhodophyta	[70,80,82,111,120,121]
Mycosporine-2-glycine<br>23427657	334	$C_{12}H_{18}N_2O_7$<br>302.11	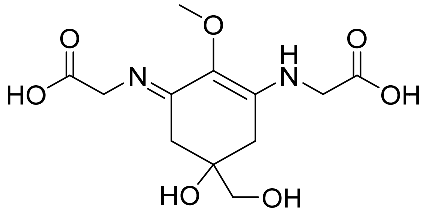<br>OC(CC(NCC(O)=O)=C/1OC)(CO)CC1=N/CC(O)=O	151.1, 164.1, 185.1, 200.1, 244.1, 288.1, <u>303.1</u>	Bacillariophyta, Cnidaria, Cyanobacteria, Echinodermata, Mollusca, Rhodophyta	[80,95,122,123]
Palythinol<br>9948334	332<br>43,500	$C_{13}H_{22}N_2O_6$<br>302.15	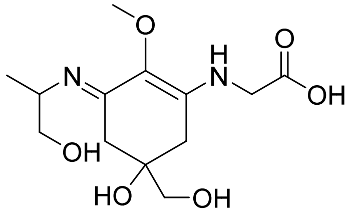<br>OC(CC(NCC(O)=O)=C/1OC)(CO)CC1=N/C(CO)C	102.2, 150.0, 186.1, 199.2, 243.2, 288.0, <u>303.5</u>	Chlorophyta, Lichen, Phaeophyta, Porifera, Rhodophyta	[70,80,83,110,117]

**Compound**<br>**Pubchem CID**	**λmax (nm)**<br>**ε ($M^{-1}.cm^{-1}$)**	**Molecular Formula**<br>**Exact mass**	**Molecular structure**<br>**Smiles**	**Fragments**	**Phylum**	**Ref.**
Asterina-methyl ester	330	$C_{13}H_{22}N_2O_6$<br>302.15	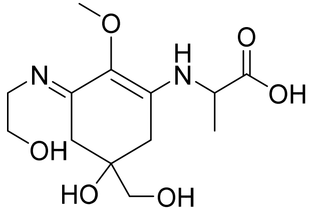<br>OC(CC(NC(C)C(O)=O)=C/1OC)(CO)C C1=N/CCO	303.2	Dinoflagellata	[124]
N-isopropanolpalythine = Aplysiapalythine A	332	$C_{13}H_{22}N_2O_6$<br>302.15	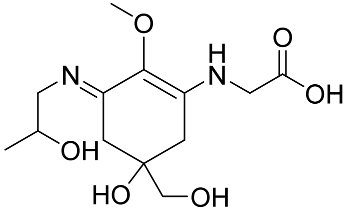<br>OC(CC(NCC(O)=O)=C/1OC)(CO)CC1= N/CC(C)O	303.2	Mollusca, Rhodophyta	[80,111]
Mycosporine-methylamine-threonine	327<br>33,300	$C_{13}H_{22}N_2O_6$<br>302.15	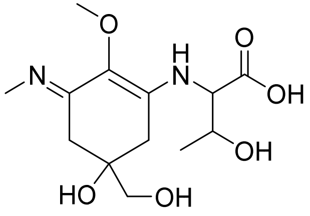<br>OC(CC(NC(C(C)O)C(O)=O)=C/1OC)(C O)CC1=N/C	303.2	Cnidaria, Rhodophyta	[80,125]

Compound<br>Pubchem CID	λmax (nm)<br>ε ($M^{-1}.cm^{-1}$)	Molecular Formula<br>Exact mass	Molecular structure<br>Smiles	Fragments	Phylum	Ref.
Palythine-glutamine<br>= Bostrychine-A	322	$C_{13}H_{21}N_3O_6$<br>315.14	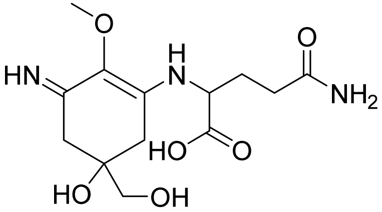<br>N=C1CC(CO)(O)CC(NC(C(O)=O)CCC(N)=O)=C1OC	316.1	Rhodophyta	[126]
Palythine-glutamic acid<br>= Bostrychine-C	322<br>22,351	$C_{13}H_{20}N_2O_7$<br>316.13	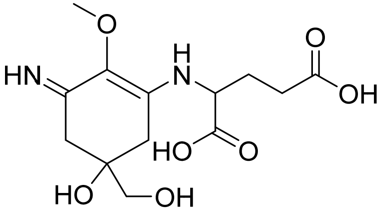<br>N=C1CC(CO)(O)CC(NC(C(O)=O)CCC(O)=O)=C1OC	317.1	Rhodophyta	[126]
Mycosporine-glycine-alanine<br>102110164	333<br>-	$C_{13}H_{20}N_2O_7$<br>316.13	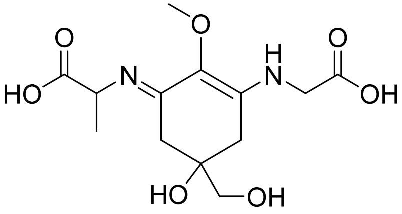<br>OC(CC(NCC(O)=O)=C/1OC)(CO)CC1=N/C(C(O)=O)C	186, 214, 258, 302, 317.1	Cyanobacteria, Rhodophyta	[80,127]

Compound<br>Pubchem CID	λmax (nm)<br>ε ($M^{-1}.cm^{-1}$)	Molecular Formula<br>Exact mass	Molecular structure<br>Smiles	Fragments	Phylum	Ref.
Palythenic acid<br>101016648	337<br>29,200	$C_{14}H_{20}N_2O_7$<br>328.13	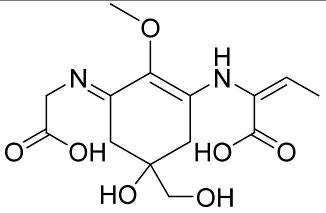<br>OC(CC(N/C(C(O)=O)=C/C)=C/1OC)(CO)CC1=N/CC(O)=O	138, 193, 197, 205, 237, 268, 283, 296, 329.1	Dinoflagellata, Mollusca, Rhodophyta	[80,81,110]
Euhalothece-362<br>102182144	362<br>-	$C_{14}H_{22}N_2O_7$<br>330.14	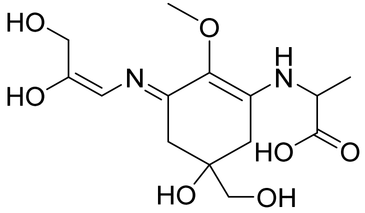<br>OC(CC(NC(C)C(O)=O)=C/1OC)(CO)CC1=N/C=C(CO)/O	213.1, 228.1, 242.1, 272.1, 316.1, 331.1	Cyanobacteria	[122,128]
Mycosporine-glutamicol ethyl ester	310<br>21,295	$C_{15}H_{25}NO_7$<br>331.16	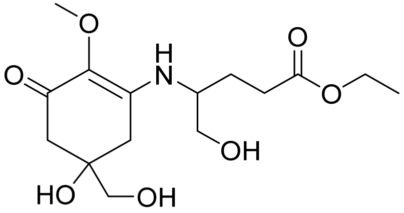<br>O=C1CC(CO)(O)CC(NC(CO)CCC(OCC)=O)=C1OC	218, 264, 286, 314, 332.2	Fungi, Lichen	[102]

Compound<br>Pubchem CID	λmax (nm)<br>ε ($M^{-1}.cm^{-1}$)	Molecular Formula<br>Exact mass	Molecular structure<br>Smiles	Fragments	Phylum	Ref.
Shinorine<br>101926676	334<br>44,668	$C_{13}H_{20}N_2O_8$<br>332.12	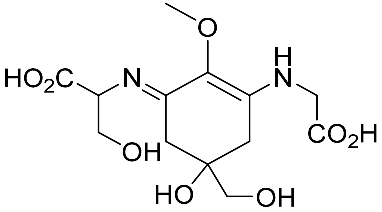<br>OC(CC(NCC(O)=O)=C/1OC)(CO)CC1=N/C(C(O)=O)CO	137.1, 186.2, 230.0, 241.3, 255.0, 298.0, 333.3	Arthropoda, Bacillariophyta, Chlorophyta, Chordata, Cyanobacteria, Dinoflagellata, Lichen, Miozoa, Mollusca, Ochrophyta, Phaeophyta, Porifera, Rhodophyta	[70,80–83,110,129]
Mycosporine-glycine-valine<br>101016647	335	$C_{15}H_{24}N_2O_7$<br>344.16	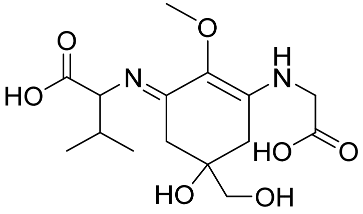<br>OC(CC(NCC(O)=O)=C/1OC)(CO)CC1=N/C(C(O)=O)C(C)C	116.0, 118.1, 130.6, 183.9, 198.1, 268.2, 295.0, 313.0	Arthropoda, Cnidaria, Chordata, Echinodermata, Lichen, Mollusca, Porifera	[70,83]
Porphyra-334<br>91864535	334<br>42,300	$C_{14}H_{22}N_2O_8$<br>346.14	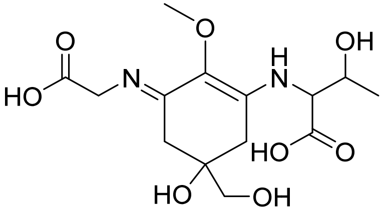<br>OC(CC(NC(C(O)C)C(O)=O)=C/1OC)(CO)CC1=N/CC(O)=O	137, 151, 168, 186, 227, 243, 288, 303, 332, 347.1	Arthropoda, Bacillariophyta, Chlorophyta, Chordata, Cnidaria, Cyanobacteria, Dinoflagellata, Miozoa, Mollusca, Ochrophyta, Phaeophyta, Porifera, Rhodophyta	[80–83,110,130]

Compound<br>Pubchem CID	λmax (nm)<br>ε (M^{-1}.cm^{-1})	Molecular Formula<br>Exact mass	Molecular structure<br>Smiles	Fragments	Phylum	Ref.
Mycosporine-serine-glycine methyl ester = Shinorine methyl ester	332<br>-	$C_{14}H_{22}N_2O_8$<br>346.14	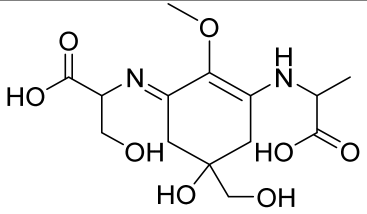<br>OC(CC(NC(C)C(O)=O)=C/1OC)(CO)CC1=N/C(C(O)=O)CO	244, 269, 288, 314, 332, 347.3	Dinoflagellata	[131]
Palythine-serine-sulfate	321<br>-	$C_{11}H_{18}N_2O_9S$<br>354.07	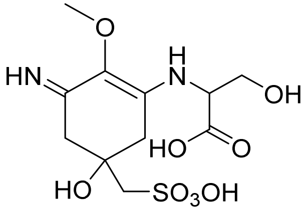<br>N=C1CC(CS(=O)(OO)=O)(O)CC(NC(CO)C(O)=O)=C1OC	355.1	Cnidaria	[120]
Mycosporine-glycine - aspartic acid	333<br>-	$C_{14}H_{20}N_2O_9$<br>360.12	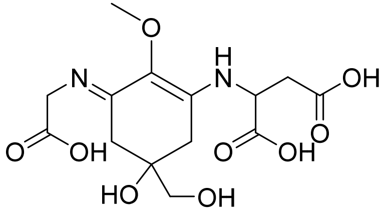<br>OC(CC(NC(CC(O)=O)C(O)=O)=C/1OC)(CO)CC1=N/CC(O)=O	361.1	Arthropoda	[75]

Compound<br>Pubchem CID	λmax (nm)<br>ε ($M^{-1}.cm^{-1}$)	Molecular Formula<br>Exact mass	Molecular structure<br>Smiles	Fragments	Phylum	Ref.
Mycosporine-threonine-β-alanine<br>= Bostrychine-F	332<br>44,994	$C_{15}H_{24}N_2O_8$<br>360.15	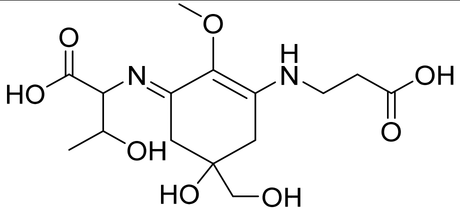<br>OC(CC(NCCC(O)=O)=C/1OC)(CO)CC1=N/C(C(C)O)C(O)=O	361.2	Rhodophyta	[126]
Palythine-threonine-sulfate	321<br>-	$C_{12}H_{20}N_2O_9S$<br>368.09	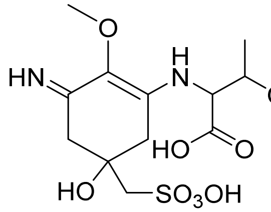<br>N=C1CC(CS(=O)(OO)=O)(O)CC(NC(C(C)O)C(O)=O)=C1OC	369.1	Cnidaria	[120]
Mycosporine-glycine - glutamic acid<br>102446627	330<br>43,900	$C_{15}H_{22}N_2O_9$<br>374.13	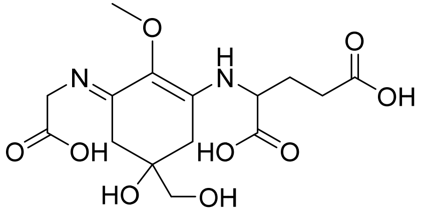<br>OC(CC(NC(CCC(O)=O)C(O)=O)=C/1OC)(CO)CC1=N/CC(O)=O	118.2, 176.0, 192.2, 228.3, 258.1, 297.1, 331.2, 375.3	Cnidaria, Lichen, Rhodophyta	[70,126,132]

Compound<br>Pubchem CID	λmax (nm)<br>ε ($M^{-1}.cm^{-1}$)	Molecular Formula<br>Exact mass	Molecular structure<br>Smiles	Fragments	Phylum	Ref.
Mycosporine-threamine-glutamic acid = Bostrychine-E	333<br>21,618	$C_{16}H_{26}N_2O_8$<br>374.17	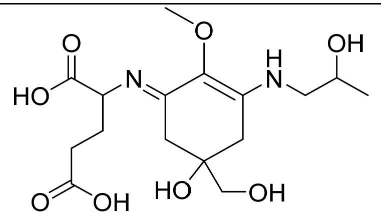<br>OC(CC(NCC(O)C)=C/1OC)(CO)CC1=N/C(CCC(O)=O)C(O)=O	375.2	Rhodophyta	[126]
Catenelline	320<br>-	$C_{13}H_{22}N_2O_9S$<br>382.10	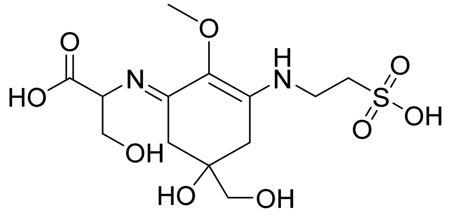<br>OC(CC(NCCS(O)(=O)=O)=C/1OC)(CO)CC1=N/C(C(O)=O)CO	383.1	Rhodophyta	[80,133]
M-343	343<br>-	-<br>386	-	387	Cyanobacteria	[96]

**Compound**<br>**Pubchem CID**	**λmax (nm)**<br>**ε ($M^{-1}.cm^{-1}$)**	**Molecular Formula**<br>**Exact mass**	**Molecular structure**<br>**Smiles**	**Fragments**	**Phylum**	**Ref.**
Mycosporine-threonine-glutamine<br>= Bostrychine-B	335<br>36,155	$C_{17}H_{27}N_3O_9$<br>417.17	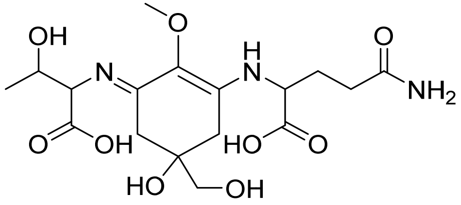<br>OC(CC(NC(C(O)=O)CCC(N)=O)=C/1O C)(CO)CC1=N/C(C(O)=O)C(O)C	418.2	Rhodophyta	[126]
Mycosporine-threonine-glutamic acid<br>= Bostrychine-D	337<br>31,956	$C_{17}H_{26}N_2O_{10}$<br>418.16	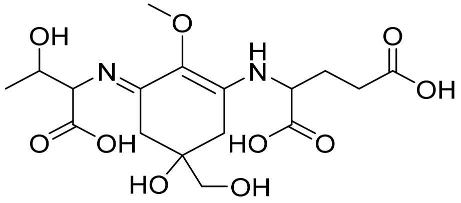<br>OC(CC(NC(C(O)=O)CCC(O)=O)=C/1O C)(CO)CC1=N/C(C(O)=O)C(O)C	419.1	Rhodophyta	[126]
**Glycosylated Oxo-mycosporines**						
Klebsormidin B	324<br>-	$C_{12}H_{19}NO_8$<br>305.11	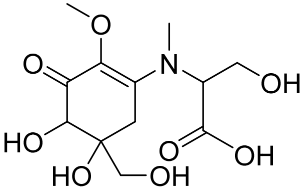<br>O=C1C(O)C(O)(CO)CC(N(C)C(C(O)=O)CO)=C1OC	306.1	Charophyta	[134]

Compound<br>Pubchem CID	λmax (nm)<br>ε ($M^{-1}.cm^{-1}$)	Molecular Formula<br>Exact mass	Molecular structure<br>Smiles	Fragments	Phylum	Ref.
Klebsormidin A	324<br>-	$C_{18}H_{29}NO_{13}$<br>467.16	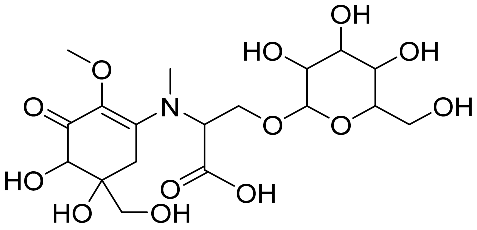<br>O=C1C(O)C(O)(CO)CC(N(C)C(C(O)=<br>O)COC2OC(CO)C(O)C(O)C2O)=C1OC	468.2	Charophyta	[134]
**Glycosylated Imino-mycosporines**						
Hexose-palythine-serine	320<br>-	$C_{17}H_{28}N_2O_{11}$<br>436.17	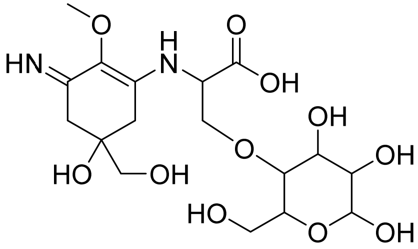<br>N=C1CC(O)(CO)CC(NC(COC2C(CO)<br>OC(O)C(O)C2O)C(O)=O)=C1OC	142, 171, 207, 239, 241, 245, 257, 275, 378, 391, 407, 422, 437.2	Cyanobacteria	[81,135]

**Compound**<br>**Pubchem CID**	**λmax (nm)**<br>**ε (M⁻¹.cm⁻¹)**	**Molecular Formula**<br>**Exact mass**	**Molecular structure**<br>**Smiles**	**Fragments**	**Phylum**	**Ref.**
Hexose-bound palythine-threonine	322<br>-	$C_{18}H_{30}N_2O_{11}$<br>450.18	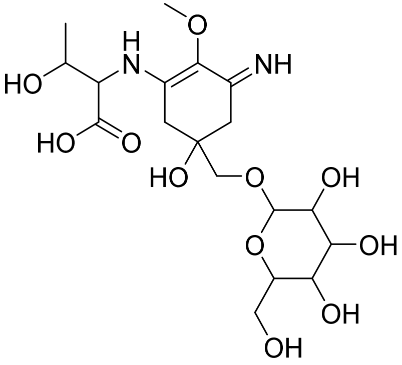<br>OC(CC(NC(C(O)=O)C(C)O)=C1OC)(COC2OC(CO)C(O)C(O)C2O)CC1=N	185.2, 289.2, 349.2, 389.2, 407.2	Cyanobacteria	[81,119]
Pentose-bound shinorine	332<br>-	$C_{18}H_{28}N_2O_{12}$<br>464.16	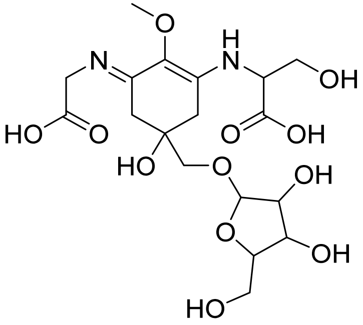<br>OC(C/1)(COC2OC(CO)C(O)C2O)CC(NC(C(O)=O)CO)=C(OC)C1=N\CC(O)=O	211.3, 333.3, 377.1, 421.1, 465.1	Cyanobacteria	[89]

Compound<br>Pubchem CID	λmax (nm)<br>ε ($M^{-1}.cm^{-1}$)	Molecular Formula<br>Exact mass	Molecular structure<br>Smiles	Fragments	Phylum	Ref.
Mycosporine-glutaminol-*O*-glucoside	310<br>25,000	$C_{19}H_{32}N_2O_{11}$<br>464.20	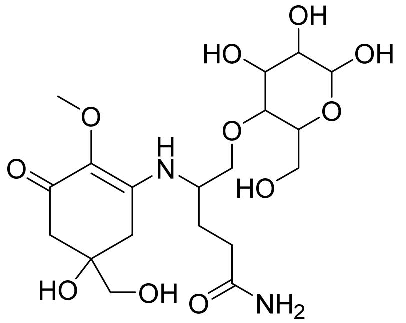<br>OC(C/1)(COC2OC(CO)C(O)C2O)CC(NC(C(O)=O)CO)=C(OC)C1=N\CC(O)=O	303, 428, 446, 465.0	Fungi	[98,136,137]
Mycosporine-glutamicol-*O*-glucoside	310<br>25,000	$C_{19}H_{31}NO_{12}$<br>465.18	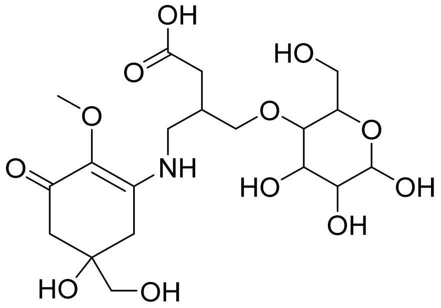<br>O=C1CC(CO)(O)CC(NCC(COC2C(O)C(O)C(O)OC2CO)CC(O)=O)=C1OC	304, 466.2	Fungi	[137,138]

Compound<br>Pubchem CID	λmax (nm)<br>ε ($M^{-1}.cm^{-1}$)	Molecular Formula<br>Exact mass	Molecular structure<br>Smiles	Fragments	Phylum	Ref.
Arabinose-bound porphyra-334 = 478 Da MAA	335<br>33,200	$C_{19}H_{30}N_2O_{12}$<br>478.18	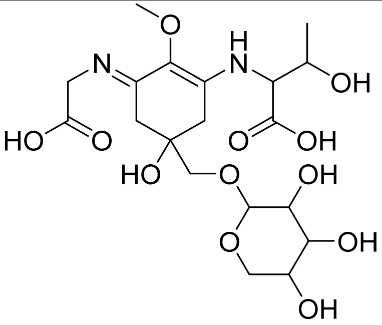<br>OC(C/1)(COC2OCC(O)C(O)C2O)CC(NC(C(O)=O)C(C)O)=C(OC)C1=N\CC(O)=O	214.8, 273.0, 377.1, 435.1, 437.1, 479.2	Cyanobacteria	[139]
Hexose-shinorine	333<br>-	$C_{19}H_{30}N_2O_{13}$<br>494.17	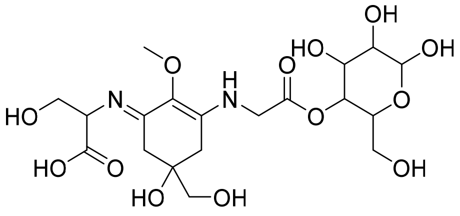<br>OC(C/1)(CO)CC(NCC(OC2C(CO)OC(O)C(O)C2O)=O)=C(OC)C1=N\C(C(O)=O)CO	186, 230, 265, 274, 287, 300, 303, 318, 333, 392, 436, 365, 480, 495.2	Cyanobacteria	[135]

Compound<br>Pubchem CID	λmax (nm)<br>ε ($M^{-1}.cm^{-1}$)	Molecular Formula<br>Exact mass	Molecular structure<br>Smiles	Fragments	Phylum	Ref.
Collemin A<br>10074531	311<br>34,000	$C_{19}H_{32}N_2O_{13}$<br>496.19	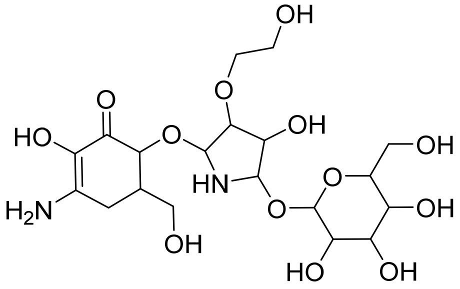<br>OC(C(C(C(CO)O1)O)O)C1OC(C2O)NC(OC3C(C(O)=C(N)CC3CO)=O)C2OCCO	95.0, 198.1, 235.8, 300.2, 340.2, 409.1, 435.0, <u>497.2</u>	Lichen	[70,140]
13-O-(β-galactosyl)-porphyra-334<br>-	334<br>-	$C_{20}H_{32}N_2O_{13}$<br>508.19	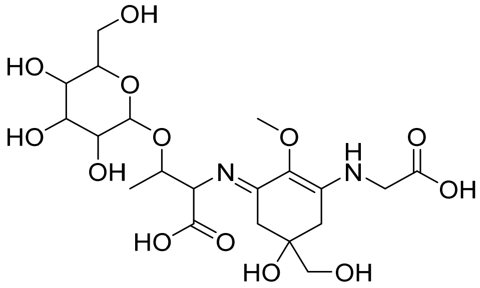<br>OC(C/1)(CO)CC(NCC(O)=O)=C(OC)C1=N\C(C(O)=O)C(OC2OC(CO)C(O)C(O)C2O)C	347.1, <u>509.2</u>	Cyanobacteria	[141]

Compound<br>Pubchem CID	λmax (nm)<br>ε (M-1.cm-1)	Molecular Formula<br>Exact mass	Molecular structure<br>Smiles	Fragments	Phylum	Ref.
Hexose-bound porphyra-334	334<br>36,300	$C_{20}H_{32}N_2O_{13}$<br>508.19	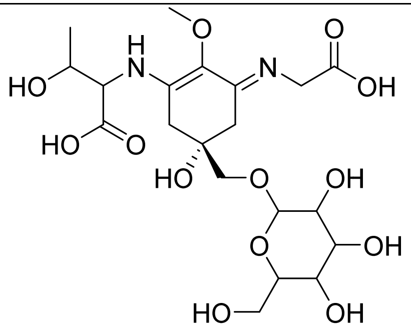<br>O[C@@](CC(NC(C(O)=O)C(C)O)=C/1OC)(COC2OC(CO)C(O)C(O)C2O)CC1=N/CC(O)=O	303.2, 347.2, 361.2, 421.2, 465.2, 509.2	Cyanobacteria	[119]
586-Da MAA	332<br>-	$C_{26}H_{42}N_4O_{11}$<br>586.29	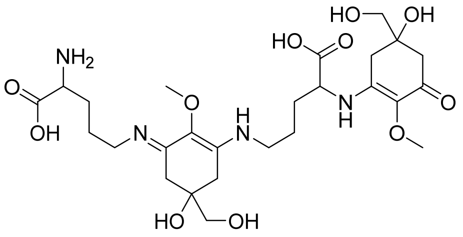<br>OC(C/1)(CO)CC(NCCCC(C(O)=O)NC2=C(OC)C(CC(CO)(O)C2)=O)=C(OC)C1=N\CCCC(N)C(O)=O	417.2, 428.2, 472.2, 499.3, 525.3, 543.3, 569.4, 587.3	Cyanobacteria	[142]

**Compound**<br>**Pubchem CID**	**λmax (nm)**<br>**ε ($M^{-1}.cm^{-1}$)**	**Molecular Formula**<br>**Exact mass**	**Molecular structure**<br>**Smiles**	**Fragments**	**Phylum**	**Ref.**
Glycosylated Palythine-threonine<br>= 612-Da MAA	324<br>28,200	$C_{24}H_{40}N_2O_{16}$<br>612.24	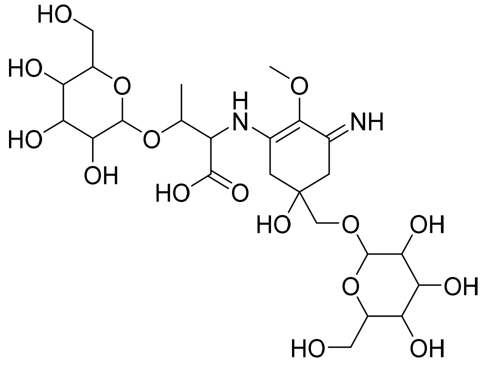<br>OC(CC(NC(C(O)=O)C(C)OC1OC(CO)C(O)C(O)C1O)=C2OC)(COC3OC(CO)C(O)C(O)C3O)CC2=N	187.2, 289.2, 349.1, 389.2, 407.2, 451.2, 569.2, 613.2	Cyanobacteria	[119]
Nostoc-756<br>= 756-Da MAA<br>146683732	313<br>-	$C_{34}H_{52}N_4O_{15}$<br>756.34	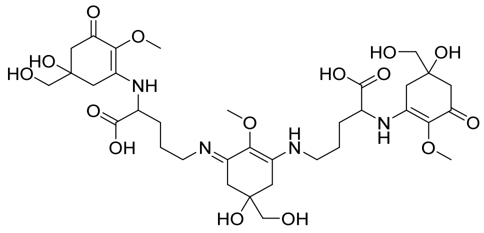<br>OC(C/1)(CO)CC(NCCCC(C(O)=O)NC2=C(OC)C(CC(CO)(O)C2)=O)=C(OC)C1=N\CCCC(NC3=C(OC)C(CC(CO)(O)C3)=O)C(O)=O	454.1, 516.1, 552.1, 603.1, 639.1, 657.1, 694.9, 757.2	Cyanobacteria	[142]

Compound<br>Pubchem CID	λmax (nm)<br>ε ($M^{-1}.cm^{-1}$)	Molecular Formula<br>Exact mass	Molecular structure<br>Smiles	Fragments	Phylum	Ref.
880-Da MAA	331<br>49,800	$C_{37}H_{60}N_4O_{20}$<br>880.38	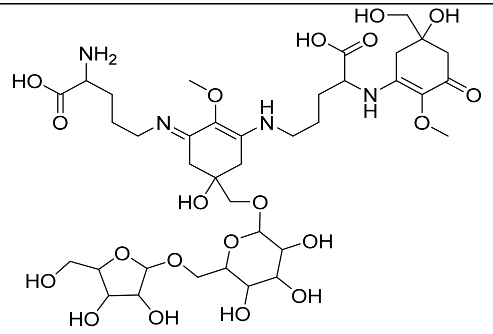<br>OC(C/1)(COC2OC(COC3OC(CO)C(O)C3O)C(O)C(O)C2O)CC(NCCCC(C(O)=O)NC4=C(OC)C(CC(CO)(O)C4)=O)=C(OC)C1=N\CCCC(N)C(O)=O	445.3, 489.3, 533.3, 737.4, 795.5, 837.5, 881.4	Cyanobacteria	[89]
1050-Da MAA	312/ 340<br>58,800	$C_{45}H_{70}N_4O_{24}$<br>1050.44	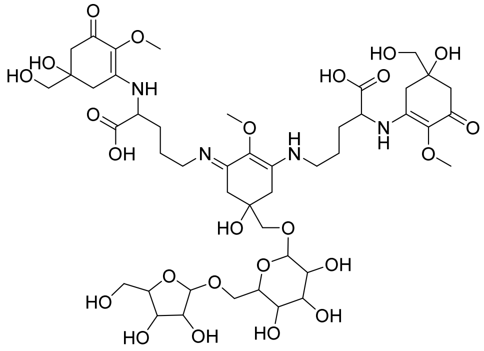<br>OC(C/1)(COC2OC(COC3OC(CO)C(O)C3O)C(O)C(O)C2O)CC(NCCCC(C(O)	410.4, 437.1, 481.1, 501.0, 581.9, 722.3, 766.3, 963.4, 1051.4	Cyanobacteria	[139]

Compound<br>Pubchem CID	λmax (nm)<br>ε ($M^{-1}.cm^{-1}$)	Molecular Formula<br>Exact mass	Molecular structure<br>Smiles	Fragments	Phylum	Ref.
			=O)NC4=C(OC)C(CC(CO)(O)C4)=O)=<br>C(OC)C1=N\CCCC(NC5=C(OC)C(CC<br>(CO)(O)C5)=O)C(O)=O			

[1] Ref: References. [a] although the correct molecular weight for dehidrousujerene is $C_{13}H_{20}N_2O_4$ (MW = 268.31) and $[M+H]^+$ might be m/z = 269, according to nitrogen rules and ionization in positive mode in ESI.

References

[1] E. Nakamura, J. Kobayashi, R. Abe, Mycosporine-Like Amino Acid, JPS59137450, 1984.

[2] G. Bird, N. Fitzmaurice, W.C. Dunlap, W.M. Chalker, B E Bandaranayake, Sunscreen compositions and compounds for use therein, International patent application PCT/AU87/00330, publication no. WO 88/02251. Australian patent 595075., 1987.

[3] G. Bird, N. Fitzmaurice, W.C. Dunlap, B.E. Chalker, W.M. Bandaranayake, Sunscreen compositions and compounds for use therein, (1991).

[4] P.J. Chalmers, N. Fitzmaurice, D.J. Rigg, S.H. Thang, G. Bird, UV-absorbing compounds and compositions, International patent application PCT/AU90/00078, publication no. WO90/09995. Australian patent 653495., 1990.

[5] M. Krol, N. HUNAR, A. Ivanov, F. Sarhan, Solar radiation protection composition, WO2000024369, 2000.

[6] N. Huner, A. Ivanov, M. Krol, F. Sarhan, Solar radiation protection composition, EP1123083A1, 2001.

[7] N. Huner, M. Krol, A. Ivanov, F. Sarhan, Solar radiation protection composition, US6787147, 2004.

[8] G. Andre, M. Pellegrini, L. Pellegrini, Algal extracts containing amino acid analogs of mycosporin are useful as dermatological protecting agents against ultraviolet radiation, FR2803201A1, 2001.

[9] D.C. Enk, M. Srebnik, O. Lev, M. Hochberg, I. Dor, A. Torres-Kerner, V. Dembitsky, Utilization of natural pigments from lichens, cyanobacteria, fungi and plants for sun protection, US20050129630A1, 2001.

[10] D.C. Enk, M. Srebnik, O. Lev, M. Hochberg, I. Dor, A. Torres-Kerner, V.M. Dembitsky, Utilization of natural pigments from lichens, cyanobacteria, fungi and plants for sun protection, WO2003020236, 2002.

[11] V.M. Dembitzky, I. Dor, D.C. Enk, M. Hochberg, O. Lev, M. Srebnik, A. Torres-Kerner, Utilization of natural pigments from lichens, cyanobacteria, fungi and plants for sun protection, AU2002329025A1, 2002.

[12] V.M. Dembitsky, I. Dor, D.C. Enk, M. Hochberg, O. Lev, M. Srebnik, A. Torres-Kerner, Utilization of natural pigments from lichens, cyanobacteria, fungi and plants for sun protection, EP1424990A2, 2002.

[13] J.C. Sirop, R.D. Pradines, Topical cosmetic composition, useful for protecting skin and hair against sunlight, contains an extract from the red alga Polysiphonia lanosa, FR2803200, 2001.

[14] C. Llewellyn, E. Galley, Personal care compositions, WO2002039974A1, 2003.

[15] C. Llewellyn, E. Galley, Personal care composotions, EP1341514A1, 2002.

[16] D.C. Enk, M. Hochberg, A. Torres, O. Lev, I. Dor, V.M. Dembitsky, M. Srebnik, Natural UV filters derived from pigments of lichens, IL0200725, 2002.

[17] N. Fujitani, M. Hori, N. Kobayashi, H. Takenaka, Y. Yamaguchi, A. Yokoyama, Extract having antioxidant activity and ultraviolet-absorbing activity, external preparation for skin, and food, JP2004238519A, 2003.

[18] D. Schmid, C. Schürch, F.D. Zülli, Cosmetic skin care products and cosmetic agents for protecting skin against premature aging, EP1473028, 2004.

[19] B. Banowski, D. Hoffmann, A. Wadle, P. Siegert, A. Saettler, T. Gerke, Beta-glucuronidase inhibitors for use in deodorants and antiperspirants, US20040234466, 2004.

[20] W. Baschong, Amino-benzophenone UV filter formulations for the prevention of tanning, GB2412866, 2005.

[21] A.J. Aguilera, L.C.L.F. De, F.F. Lopez, Use of a mycosporin-type amino acid (porphyra 334) as an antioxidant, WO2007026035, 2005.

[22] J.A. Arjona, F.D.L.C. Luque, F.L. Figueroa, Use of a mycosporin-type amino acid (porphyra 334) as an antioxidant, ES2301293, 2005.

[23] F.D.L.C. Luque, J.A. ARJONA, F.L. FIGUEROA, Use of a mycosporin-type amino acid (shinorine) as an antioxidant, WO2007026038, 2005.

[24] J.A. Arjona, F.D.L.C. Luquea, F.L. Figueroa, Use of a mycosporin-type amino acid (shinorine) as an antioxidant, ES2301426, 2005.

[25] J.A. Arjona, F.D.L.C. Luque, F.L. Figueroa, Use of a mixture of mycosporin-type amino acids (asterin 330 + palythine) as an antioxidant, WO2007026037, 2005.

[26] A.J. Aguilera, L.C.L.F. De, F.F. Lopez, Use of a mycosporin-type amino acid (m-gly) as an antioxidant, WO2007026036A3, 2006.

[27] H.S. Ewart, J. Zhang, J.C. Barrow, Compositions comprising porphyra and methods of making and using thereof, WO2007144779, 2007.

[28] H.S. Ewart, J. Zhang, J.C. Barrow, Compositions comprising porphyra and methods of making and using thereof, EP2001311, 2007.

[29] H. Ewart, J. Zhang, C. Barrow, Compositions comprising Porphyra and methods of making and using thereof, US20070220806, 2006.

[30] J.A. Arjona, F.D.L.C. Luque, N.K. Peinado, F.L. Figueroa, Composition for solar protection based on algae and liquenes extracts. (Machine-translation by Google Translate, not legally binding), ES2317741, 2006.

[31] S. Benedetti, F. Canestrari, S. Scoglio, L. Zolla, Extracts of aphanizomenon flos aquae and nutritional, cosmetic and pharmaceutical compositions containing the same, WO2008000431, 2007.

[32] S. Scoglio, F. Canestrari, S. Benedetti, L. Zolla, Extracts of aphanizomenon flos aquae (afa klamath), active compounds, and their uses, EP2032122, 2007.

[33] S. Scoglio, F. Canestrari, S. Benedetti, L. Zolla, Extracts of aphanizomenon flos aquae and nutritional, cosmetic and pharmaceutical compositons containing the same, US20100021493, 2007.

[34] S. Scoglio, F. Canestrari, S. Benedetti, Y. Benedetti, M. Delgado-Esteban, Alphanizomenon flos aquae preparation, extracts and purified components thereof for the treatment

of neurological, neurodegenerative and mood disorders, WO2008000430, 2007.

[35] S. Benedetti, Y. Benedetti, F. Canestrari, M. Delgado-Esteban, S. Scoglio, Alphanizomenon flos aquae preparation, extracts and purified components thereof for the treatment of neurological, neurodegenerative and mood disorders, EP2046354, 2007.

[36] S. Scoglio, F. Canestrari, S. Benedetti, Y. Benedetti, M. Delgado-Esteban, Alphanizomenon flos aquae preparation, extracts and purified components thereof for the treatment of neurological, neurodegenerative and mood disorders, US20090311286, 2007.

[37] M. Miyamoto, M. Sakakibara, M. Torii, Mycosporin-like amino acid derivative having glycosyl group and method for producing the same, JP2009120562, 2007.

[38] Gao Kunshan, Li Ping, Xu Juntian, Chen Zhihui, Qiu Yuming, Cosmetic including natural substance having sun-screening function, CN101061995, 2007.

[39] S. Abe, K. Koganemaru, K. Tsuge, T. Tsuruhashi, H. Tsuruta, M. Yoshiki, T. Yoshimura, Antioxidant compound, antioxidant algae extract and method for producing the same, JP2008247901A, 2008.

[40] F. Wolf, Cosmetic sunscreen composition, GB2472021, 2009.

[41] Taejun Han, Park Jin-hee, Method for preparing uv screening nontoxic extract from red algae, and nontoxic sunscreen using same, WO2011096628, 2010.

[42] C. O’connor, S.C. Skill, C.A. Llewellyn, Topical composition, WO2011158041, 2011.

[43] Z. Chaohui, Gao Xin, Xu Zhiheng, Xu Jiachao, Preparation method for laver mycosporine-like amino acids Porphyra-334, CN102659621, 2012.

[44] Su Zhenhong, Lin Jing, Wang Fang, Beauty product containing desert algae radiation-proof ingredient and natural medical whitening ingredient and preparation method thereof, CN102764206, 2012.

[45] E. Abou-Khalil, S. RAEPPEL, F. Raeppel, Imino compounds as protecting agents against ultraviolet radiations, WO2013181741, 2013.

[46] E. Abou-Khalil, S. Raeppel, F. Raeppel, Imino compounds as protecting agents againts ultraviolet radiations, US20150152046, 2013.

[47] K. Ishihara, R. Watanabe, T. Suzuki, T. Sakamoto, S. Matsugo, N. Wada, H. Takenaka, Y. Yamaguchi, Mycosporine-like amino acid and method for producing the same, UV protection agent, and antioxidant, JP6049200B2, 2013.

[48] K. Ishihara, R. Watanabe, T. Suzuki, T. Sakamoto, S. Matsusato, N. Wada, H. Takenaka, Y. Yamaguchi, Mycosporin-like amino acids, production method thereof, uv protecting agents and antioxidants, JP2014227339, 2013.

[49] Yu Gongliang, Li Renhui, A kind of method extracting class mycetocyte element amino acid Shinorine from Microcystis aeruginosa, CN103755589, 2013.

[50] N. QVIT-RAZ, T. ALTMAN, Topical composition comprising transformed bacteria expressing a compound of interest, WO2014025938, 2013.

[51] N. Qvit-Raz, T. Altman, Topical composition comprising transformed bacteria expressing a compound of interest, US20160000701, 2015.

[52] J. Ryan, M. York, Synthesis of uv absorbing compounds, WO2014082124, 2013.

[53] M. York, J. Ryan, Synthesis of uv absorbing compounds, US20150299124, 2013.

[54] Mo Sang-hyun, Chong Tae-Hyon, Jeonghoon Lee, Seo Hyo-hyun, Jeong Haesoo, Moonjin Cho, Hyun Lee, Shin Dongseon, Lee Yoo-ri, Binary, Kim Hyung Sik, Anti-UV composition for skin external application comprising peptide derivatives from extract of microalgae comprising the same, KR20150008238A, 2013.

[55] M. York, J. Ryan, G.P. SAVAGE, A.G. Meyer, K. Jarvis, UV absorbing compounds, compositions comprising same and uses thereof, WO2015006803, 2014.

[56] M. York, J. Ryan, G.P. SAVAGE, A.G. Meyer, K. Jarvis, UV absorbing compounds, compositions comprising same and uses thereof, US20160244409, 2014.

[57] N. QVIT-RAZ, Topical formulations for uv protection, WO2015195546, 2015.

[58] H. Ikeda, Y. Shogo, M. Atsushi, M. Sota, Method for producing mycosporine-like amino acid using microbes, WO2015174427, 2015.

[59] H. Ikeda, S. Yamamoto, J. Matsumoto, M. Sota, Method for producing mycosporine-like amino acid using microbes, US2017202762A1, 2015.

[60] H. Ikeda, S. Yamamoto, J. Matsumoto, M. Sota, Method for producing mycosporine-like amino acid using microbes, EP3144392A1, 2015.

[61] K. Matsuyama, Y. Shogo, Novel mycosporine-like amino acid, WO2017082144A1, 2016.

[62] Kiwoo Kim, Yang Dongju, Mo Sang-hyun, Yoonhee Choi, Seo Hyo-hyun, Jeonghoon Lee, Kim Hyung Sik, Wound healing composition for skin external application comprising Mycosporine-like amino acid and Method for Preparing the Same, KR20170090690A, 2016.

[63] Zhou Haomiao, Bi Tianyu, Liu Li, Yang Panpan, Bi Yongxian, Jiang Ligang, Culture method capable of improving mycosporine-like amino acid content in umbilical laver, CN105684880B, 2016.

[64] A. Yamamoto, K. Nakamura, K. Morotomi, Extraction of mycosporine-like amino acids consisting of amino acid derivatives, separation and purification with chemically modified surface-modified activated carbon filler, and automation of their production, JP6058853B1, 2016.

[65] S. Kim, K. Lee, J.H. Lee, J. SEOK, J.W. Jang, Microorganism for producing a mycosporine-like amino acid method for producing a mycosporine-like amino acid using the same, CA3072748A1, 2017.

[66] Kim Sol, Seok Jong-cheol, Gyuseong Lee, Jaewoo Jang, A microorganism for producing a Mycosporine-like amino acid and a method for preparing a Mycosporine-like amino acid using the same, KR102003911B1, 2018.

[67] F. ULICZKA-OPITZ, M. SCHOTTKOWSKI, U. Duhring, Production of mycosporine-like amino acids in cyanobacteria, WO2019094447A2, 2018.

[68] S. Kim, K. LEE, J.H. Lee, J. Seok, J. woo Jang, Microorganism for producing a mycosporine-like amino acid and method for producing a mycosporine-like amino acid using the same, US20200283810A1, 2020.

[69] A. Matsumoto, K. Matsuyama, S. Yamamoto, K. Kanazawa, Solution containing stabilized mycosporine-like amino acid and method for producing the same, JP2020114871A, 2020.

[70] V. Shukla, R. Kumari, D.K. Patel, D.K. Upreti, Characterization of the diversity of mycosporine-like amino acids in lichens from high altitude region of Himalaya, Amino Acids. 48 (2016) 129–136. doi:10.1007/s00726-015-2069-z.

[71] S. Ito, Y. Hirata, Isolation and structure of a mycosporine from the zoanthid Palythoa tuberculosa, Tetrahedron Lett. 18 (1977) 2429–2430. doi:10.1016/S0040-4039(01)83784-9.

[72] F. Chioccara, A. Delia Gala, M. De Rosa, E. Novellino, G. Prota, Mycosporine aminoacids and related compounds from the eggs of fishes, Bull. Des Sociétés Chim. Belges. 89 (2010) 1101–1106. doi:10.1002/bscb.19800891212.

[73] F. Chioccara, L. Zeuli, E. Novellino, Occurrence of mycosporine related compounds in sea urchin eggs, Comp. Biochem. Physiol. Part B Comp. Biochem. 85 (1986) 459–461. doi:10.1016/0305-0491(86)90027-1.

[74] P.A. Plack, N.W. Fraser, P.T. Grant, C. Middleton, A.I. Mitchell, R.H. Thomson, Gadusol, an enolic derivative of cyclohexane-1,3-dione present in the roes of cod and other marine fish. Isolation, properties and occurrence compared with ascorbic acid, Biochem. J. 199 (1981) 741–747. doi:10.1042/bj1990741.

[75] P.T. Grant, C. Middleton, P.A. Plack, R.H. Thomson, The isolation of four aminocyclohexenimines (mycosporines) and a structurally related derivative of cyclohexane-1:3-dione (gadusol) from the brine shrimp, Artemia, Comp. Biochem. Physiol. Part B Comp. Biochem. 80 (1985) 755–759. doi:10.1016/0305-0491(85)90457-2.

[76] P.T. Grant, P.A. Plack, R.H. Thomson, Gadusol, a metabolite from fish eggs, Tetrahedron Lett. 21 (1980) 4043–4044. doi:10.1016/S0040-4039(00)92866-1.

[77] Y. Oda, Q. Zhang, S. Matsunaga, M.J. Fujita, R. Sakai, Two new mycosporine-like amino acids LC-343 and mycosporine-ethanolamine from the micronesian marine sponge Lendenfeldia chondrodes, Chem. Lett. 46 (2017) 1272–1274. doi:10.1246/cl.170490.

[78] J.D. White, J.H. Cammack, K. Sakuma, The synthesis and absolute configuration of mycosporins. A novel application of the Staudinger reaction, J. Am. Chem. Soc. 111 (1989) 8970–8972. doi:10.1021/ja00206a059.

[79] C. Oyamada, M. Kaneniwa, K. Ebitani, M. Murata, K. Ishihara, Mycosporine-like amino acids extracted from scallop (Patinopecten yessoensis) ovaries: UV protection and growth stimulation activities on human cells, Mar. Biotechnol. 10 (2008) 141–150. doi:10.1007/s10126-007-9043-z.

[80] Y. Sun, N. Zhang, J. Zhou, S. Dong, X. Zhang, L. Guo, G. Guo, Distribution, contents, and types of mycosporine-like amino acids (MAAs) in marine macroalgae and a database for MAAs based on these characteristics, Mar. Drugs. 18 (2020) 43. doi:10.3390/md18010043.

[81] V. Geraldes, F.R. Jacinavicius, D.B. Genuário, E. Pinto, Identification and distribution of mycosporine-like amino acids in Brazilian cyanobacteria using ultrahigh-performance liquid chromatography with diode array detection coupled to quadrupole time-of-flight mass spectrometry, Rapid Commun. Mass Spectrom. (2020). doi:10.1002/rcm.8634.

[82] S. Jeffrey, H. MacTavish, W. Dunlap, M. Vesk, K. Groenewoud, Occurrence of UVA- and UVB-absorbing compounds in 152 species (206 strains) of marine microalgae, Mar. Ecol. Prog. Ser. 189 (1999) 35–51. doi:10.3354/meps189035.

[83] D. Karentz, F.S. McEuen, M.C. Land, W.C. Dunlap, Survey of mycosporine-like amino acid compounds in Antarctic marine organisms: Potential protection from ultraviolet exposure, Mar. Biol. 108 (1991) 157–166. doi:10.1007/BF01313484.

[84] B. Leite, R.L. Nicholson, Mycosporine-alanine: A self-inhibitor of germination from the conidial mucilage of Colletotrichum graminicola, Exp. Mycol. 16 (1992) 76–86. doi:10.1016/0147-5975(92)90043-Q.

[85] N. Wada, T. Sakamoto, S. Matsugo, Mycosporine-Like Amino Acids and Their Derivatives as Natural Antioxidants, Antioxidants. 4 (2015) 603–646. doi:10.3390/antiox4030603.

[86] E. Nakamura, J. Kobayashi, R. Abe, Mycosporin-Like Amino Acid, JP 1984137450 A, 1984.

[87] J.I. Carreto, S. Roy, K. Whitehead, C.A. Llewellyn, M.O. Carignan, UV-absorbing 'pigments': mycosporine-like amino acids, in: Phytoplankt. Pigment., 2012: pp. 412–442. doi:10.1017/cbo9780511732263.015.

[88] C. Roullier, M. Chollet-Krugler, A. Bernard, J. Boustie, Multiple dual-mode centrifugal partition chromatography as an efficient method for the purification of a mycosporine from a crude methanolic extract of Lichina pygmaea, J. Chromatogr. B. 877 (2009) 2067–2073. doi:10.1016/j.jchromb.2009.05.040.

[89] E. Nazifi, N. Wada, T. Asano, T. Nishiuchi, Y. Iwamuro, S. Chinaka, S. Matsugo, T. Sakamoto, Characterization of the chemical diversity of glycosylated mycosporine-like amino acids in the terrestrial cyanobacterium Nostoc commune, J. Photochem. Photobiol. B Biol. 142 (2015) 154–168. doi:10.1016/j.jphotobiol.2014.12.008.

[90] E. Nazifi, N. Wada, T. Asano, T. Nishiuchi, Y. Iwamuro, S. Chinaka, S. Matsugo, T. Sakamoto, Corrigendum to "Characterization of the chemical diversity of glycosylated mycosporine-like amino acids in the terrestrial cyanobacterium Nostoc commune" [J. Photochem. Photobiol. B: Biol. 142 (2015) 154–168], J. Photochem. Photobiol. B Biol. 144 (2015) 75. doi:10.1016/j.jphotobiol.2015.02.004.

[91] N. Arpin, Mycosporines: review and new data concerning their structure, Rev. Mycol. 43 (1979) 247–257.

[92] M.-C. Lunel, N. Arpin, J. Favre-Bonvin, Structure de la Nor-Mycosporine Glutamine, nouvelle substance isolee de (Bull ex Fr.) fuckel, Tetrahedron Lett. 21 (1980) 4715–4716.

doi:10.1016/0040-4039(80)88101-9.

[93] N. Arpin, Structure de la mycosporine 2, nouvelle molecule, isolee de Botrytis cinerea, (1977).

[94] J. Fayret, J. Bernillon, M.-L. Bouillant, J. Favre-Bonvin, N. Arpin, Open and ring forms of mycosporin-2 from the ascomycete Gnomonia leptostyla, Phytochemistry. 20 (1981) 2709–2710. doi:10.1016/0031-9422(81)85273-9.

[95] W.R. Stochaj, W.C. Dunlap, J.M. Shick, Two new UV-absorbing mycosporine-like amino acids from the sea anemoneAnthopleura elegantissima and the effects of zooxanthellae and spectral irradiance on chemical composition and content, Mar. Biol. 118 (1994) 149–156. doi:10.1007/BF00699229.

[96] L. Zhang, L. Li, Q. Wu, Protective effects of mycosporine-like amino acids of Synechocystis sp. PCC 6803 and their partial characterization, J. Photochem. Photobiol. B Biol. 86 (2007) 240–245. doi:10.1016/j.jphotobiol.2006.10.006.

[97] J. Pathak, R. Richa, A.S. Sonker, V.K. Kannaujiya, R.P. Sinha, Isolation and partial purification of scytonemin and mycosporine-like amino acids from biological crusts, J. Chem. Pharm. Res. 7 (2015) 362–371.

[98] J. Bernillon, M.-L. Bouillant, J.-L. Pittet, J. Favre-Bonvin, N. Arpin, Mycosporine glutamine and related mycosporines in the fungus Pyronema omphalodes, Phytochemistry. 23 (1984) 1083–1087. doi:10.1016/S0031-9422(00)82614-X.

[99] M. Katoch, R. Mazmouz, R. Chau, L.A. Pearson, R. Pickford, B.A. Neilan, Heterologous production of cyanobacterial mycosporine-like amino acids mycosporine-ornithine and mycosporine-lysine in Escherichia coli, Appl. Environ. Microbiol. 82 (2016) 6167–6173. doi:10.1128/AEM.01632-16.

[100] F. Lemoyne, J. Bernillon, J. Favre-Bonvin, M.L. Bouillant, N. Arpin, Occurrence and characteristics of amino alcohols and cyclohexenone. Components of fungal mycosporines, Zeitschrift Für Naturforsch. C. 40 (1985) 612–616. doi:10.1515/znc-1985-9-1004.

[101] J. Favre-Bonvin, J. Bernillon, N. Salin, N. Arpin, Biosynthesis of mycosporines: Mycosporine glutaminol in Trichothecium roseum, Phytochemistry. 26 (1987) 2509–2514. doi:10.1016/S0031-9422(00)83866-2.

[102] T. Nguyen, M. Chollet-Krugler, F. Lohézic-Le Dévéhat, I. Rouaud, J. Boustie, Mycosporine-like compounds in chlorolichens: isolation from Dermatocarpon luridum and

Dermatocarpon miniatum, and their photoprotective properties, Planta Medica Lett. 2 (2015) e1–e5. doi:10.1055/s-0034-1396321.

[103] C. Roullier, M. Chollet-Krugler, E.-M. Pferschy-Wenzig, A. Maillard, G.N. Rechberger, B. Legouin-Gargadennec, R. Bauer, J. Boustie, Characterization and identification of mycosporines-like compounds in cyanolichens. Isolation of mycosporine hydroxyglutamicol from Nephroma laevigatum Ach., Phytochemistry. 72 (2011) 1348–1357. doi:10.1016/j.phytochem.2011.04.002.

[104] F. Garcia-Pichel, R.W. Castenholz, Occurrence of UV-absorbing, mycosporine-like compounds among cyanobacterial isolates and an estimate of their screening capacity, Appl. Environ. Microbiol. 59 (1993) 163–169. doi:10.1128/AEM.59.1.163-169.1993.

[105] H. Young, V.J. Patterson, A UV protective compound from Glomerella cingulata—a mycosporine, Phytochemistry. 21 (1982) 1075–1077. doi:10.1016/S0031-9422(00)82419-X.

[106] A. Hartmann, A. Holzinger, M. Ganzera, U. Karsten, Prasiolin, a new UV-sunscreen compound in the terrestrial green macroalga Prasiola calophylla (Carmichael ex Greville) Kützing (Trebouxiophyceae, Chlorophyta), Planta. 243 (2016) 161–169. doi:10.1007/s00425-015-2396-z.

[107] K.H.M. Cardozo, L.G. Marques, V.M. Carvalho, M.O. Carignan, E. Pinto, E. Marinho-Soriano, P. Colepicolo, Analyses of photoprotective compounds in red algae from the Brazilian coast, Rev. Bras. Farmacogn. 21 (2011) 202–208. doi:10.1590/S0102-695X2011005000047.

[108] S. Takano, D. Uemura, Y. Hirata, Isolation and structure of a new amino acid, palythine, from the zoanthid, Tetrahedron Lett. 19 (1978) 2299–2300. doi:10.1016/S0040-4039(01)91519-9.

[109] I. Tsujino, K. Yabe, I. Sekikawa, N. Hamanaka, Isolation and structure of a mycosporine from the red alga, Tetrahedron Lett. 19 (1978) 1401–1402. doi:10.1016/S0040-4039(01)94556-3.

[110] W. K., K. D., H. J., Mycosporine-like amino acids (MAAs) in phytoplankton, a herbivorous pteropod (Limacina helicina), and its pteropod predator (Clione antarctica) in McMurdo Bay, Antarctica, Mar. Biol. 139 (2001) 1013–1019. doi:10.1007/s002270100654.

[111] M. Kamio, C.E. Kicklighter, L. Nguyen, M.W. Germann, C.D. Derby, Isolation and structural elucidation of novel mycosporine-like amino acids as alarm cues in the defensive ink secretion of the sea hare Aplysia californica, Helv. Chim. Acta. 94 (2011) 1012–1018. doi:10.1002/hlca.201100117.

[112] N. Werner, M. Orfanoudaki, A. Hartmann, M. Ganzera, R. Sommaruga, Low temporal dynamics of mycosporine-like amino acids in benthic cyanobacteria from an alpine lake, Freshw. Biol. 66 (2021) 169–176. doi:10.1111/fwb.13627.

[113] M. Orfanoudaki, A. Hartmann, U. Karsten, M. Ganzera, Chemical profiling of mycosporine-like amino acids in twenty-three red algal species, J. Phycol. 55 (2019) 393–403. doi:10.1111/jpy.12827.

[114] T.T. Teai, P. Raharivelomanana, J.-P. Bianchini, R. Faure, P.M.. Martin, A. Cambon, Structure de deux nouvelles iminomycosporines isolées de Pocillopora eydouxi, Tetrahedron Lett. 38 (1997) 5799–5800. doi:10.1016/S0040-4039(97)01281-1.

[115] I. Sekikawa, Isolation and structure of a 357 nm UV-absorbing substance, usujirene, from the red alga Palmaria palmata (L.) O. Kuntze, Jpn. J. Phycol. 34 (1986) 185–188.

[116] K.H.M. Cardozo, Estudos de compostos fotoprotetores da radiaçao ultravioleta em algas: Aminoácidos tipo micosporinas (MAAs), Universidade de São Paulo, 2007.

[117] S. Takano, D. Uemura, Y. Hirata, Isolation and structure of two new amino acids, palythinol and palythene, from the zoanthid, Tetrahedron Lett. 19 (1978) 4909–4912. doi:10.1016/S0040-4039(01)85768-3.

[118] M.O. Carignan, K.H.M. Cardozo, D. Oliveira-Silva, P. Colepicolo, J.I. Carreto, Palythine–threonine, a major novel mycosporine-like amino acid (MAA) isolated from the hermatypic coral Pocillopora capitata, J. Photochem. Photobiol. B Biol. 94 (2009) 191–200. doi:10.1016/j.jphotobiol.2008.12.001.

[119] E. Nazifi, N. Wada, M. Yamaba, T. Asano, T. Nishiuchi, S. Matsugo, T. Sakamoto, Glycosylated Porphyra-334 and Palythine-Threonine from the Terrestrial Cyanobacterium Nostoc commune, Mar. Drugs. 11 (2013) 3124–3154. doi:10.3390/md11093124.

[120] J.J. Wu Won*, B.E. Chalker, J.A. Rideout, Two new UV-absorbing compounds from Stylophora pistillata: sulfate esters of mycosporine-like amino acids, Tetrahedron Lett. 38 (1997) 2525–2526. doi:10.1016/S0040-4039(97)00391-2.

[121] H. Nakamura, J. Kobayashi, Y. Hirata, Isolation and structure of a 330 nm UV-absorbing substance, asterina-330 from the starfish Asterina pectinifera, Chem. Lett. 10 (1981) 1413–1414. doi:10.1246/cl.1981.1413.

[122] L. Kedar, Y. Kashman, A. Oren, Mycosporine-2-glycine is the major mycosporine-like amino acid in a unicellular cyanobacterium (Euhalothece sp.) isolated from a gypsum

crust in a hypersaline saltern pond, FEMS Microbiol. Lett. 208 (2002) 233–237. doi:10.1111/j.1574-6968.2002.tb11087.x.

[123] J. Shick, W. Dunlap, B. Chalker, A. Banaszak, T. Rosenzweig, Survey of ultraviolet radiation-absorbing mycosporine-like amino acids in organs of coral reef holothuroids, Mar. Ecol. Prog. Ser. 90 (1992) 139–148. doi:10.3354/meps090139.

[124] D.A. White, L. Polimene, C.A. Llewellyn, Effects of ultraviolet-a radiation and nutrient availability on the cellular composition of photoprotective compounds in glenodinium foliaceum (dinophyceae)1, J. Phycol. 47 (2011) 1078–1088. doi:10.1111/j.1529-8817.2011.01046.x.

[125] J.J. Wu Won, J.A. Rideout, B.E. Chalker, Isolation and structure of a novel mycosporine-like amino acid from the reef-building corals Pocillopora damicornis and Stylophora pistillata, Tetrahedron Lett. 36 (1995) 5255–5256. doi:10.1016/0040-4039(95)00950-H.

[126] M. Orfanoudaki, A. Hartmann, H. Miladinovic, H. Nguyen Ngoc, U. Karsten, M. Ganzera, Bostrychines A–F, six novel mycosporine-like amino-acids and a novel Betaine from the red alga Bostrychia scorpioides, Mar. Drugs. 17 (2019) 356. doi:10.3390/md17060356.

[127] V. Geraldes, L.S. de Medeiros, S.T. Lima, D.O. Alvarenga, R. Gacesa, P.F. Long, M.F. Fiore, E. Pinto, Genetic and biochemical evidence for redundant pathways leading to mycosporine-like amino acid biosynthesis in the cyanobacterium Sphaerospermopsis torques-reginae ITEP-024, ALGAE. 35 (2020) 177–187. doi:10.4490/algae.2020.35.5.19.

[128] M. Volkmann, A.A. Gorbushina, L. Kedar, A. Oren, Structure of euhalothece-362, a novel red-shifted mycosporine-like amino acid, from a halophilic cyanobacterium (Euhalothece sp.), FEMS Microbiol. Lett. 258 (2006) 50–54. doi:10.1111/j.1574-6968.2006.00203.x.

[129] I. Tsujino, Isolation and structure of a new amino acid, shinorine, from the red alga Chondrus yendoi Yamada et Mikami, (1980).

[130] Richa, R.P. Sinha, Biochemical characterization of sunscreening mycosporine-like amino acids from two Nostoc species inhabiting diverse habitats, Protoplasma. 252 (2015) 199–208. doi:10.1007/s00709-014-0674-4.

[131] M.O. Carignan, J.I. Carreto, Characterization of mycosporine-serine-glycine methyl ester, a major mycosporine-like amino acid from dinoflagellates: a mass spectrometry study, J. Phycol. 49 (2013) 680–688. doi:10.1111/jpy.12076.

[132] W.M. Bandaranayake, J.E. Bemis, D.J. Bourne, Ultraviolet absorbing pigments from the marine sponge Dysidea herbacea: Isolation and structure of a new mycosporine,

Comp. Biochem. Physiol. Part C Pharmacol. Toxicol. Endocrinol. 115 (1996) 281–286. doi:10.1016/S0742-8413(96)00135-1.

[133] A. Hartmann, K. Becker, U. Karsten, D. Remias, M. Ganzera, Analysis of mycosporine-like amino acids in selected algae and cyanobacteria by hydrophilic interaction liquid chromatography and a novel MAA from the red alga Catenella repens, Mar. Drugs. 13 (2015) 6291–6305. doi:10.3390/md13106291.

[134] A. Hartmann, K. Glaser, A. Holzinger, M. Ganzera, U. Karsten, Klebsormidin A and B, Two New UV-Sunscreen Compounds in Green Microalgal Interfilum and Klebsormidium Species (Streptophyta) From Terrestrial Habitats, Front. Microbiol. 11 (2020) 499. doi:10.3389/fmicb.2020.00499.

[135] P.M. D'Agostino, V.S. Javalkote, R. Mazmouz, R. Pickford, P.R. Puranik, B.A. Neilan, Comparative profiling and discovery of novel glycosylated mycosporine-Like amino acids in two strains of the cyanobacterium Scytonema cf. crispum, Appl. Environ. Microbiol. 82 (2016) 5951–5959. doi:10.1128/AEM.01633-16.

[136] R. Sommaruga, D. Libkind, M. van Broock, K. Whitehead, Mycosporine-glutaminol-glucoside, a UV-absorbing compound of two Rhodotorula yeast species, Yeast. 21 (2004) 1077–1081. doi:10.1002/yea.1148.

[137] M. Volkmann, K. Whitehead, H. Rütters, J. Rullkötter, A.A. Gorbushina, Mycosporine-glutamicol-glucoside: a natural UV-absorbing secondary metabolite of rock-inhabiting microcolonial fungi, Rapid Commun. Mass Spectrom. 20 (2006) 2520–2520. doi:10.1002/rcm.2616.

[138] M.-L. Bouillant, J.-L. Pittet, J. Bernillon, J. Favre-Bonvin, N. Arpin, Mycosporins from Ascochyta pisi, Cladosporium herbarum and Septoria nodorum, Phytochemistry. 20 (1981) 2705–2707. doi:10.1016/0031-9422(81)85272-7.

[139] K. Matsui, E. Nazifi, S. Kunita, N. Wada, S. Matsugo, T. Sakamoto, Novel glycosylated mycosporine-like amino acids with radical scavenging activity from the cyanobacterium Nostoc commune, J. Photochem. Photobiol. B Biol. 105 (2011) 81–89. doi:10.1016/j.jphotobiol.2011.07.003.

[140] A. Torres, M. Hochberg, I. Pergament, R. Smoum, V. Niddam, V.M. Dembitsky, M. Temina, I. Dor, O. Lev, M. Srebnik, C.D. Enk, A new UV-B absorbing mycosporine with photo protective activity from the lichenized ascomycete Collema cristatum, Eur. J. Biochem. 271 (2004) 780–784. doi:10.1111/j.1432-1033.2004.03981.x.

[141] K. Ishihara, R. Watanabe, H. Uchida, T. Suzuki, M. Yamashita, H. Takenaka, E. Nazifi, S. Matsugo, M. Yamaba, T. Sakamoto, Novel glycosylated mycosporine-like amino acid, 13- O -(β-galactosyl)-porphyra-334, from the edible cyanobacterium Nostoc sphaericum -protective activity on human keratinocytes from UV light, J. Photochem. Photobiol. B Biol. 172 (2017) 102–108. doi:10.1016/j.jphotobiol.2017.05.019.

[142] T. Sakamoto, A. Hashimoto, M. Yamaba, N. Wada, T. Yoshida, K. Inoue-Sakamoto, T. Nishiuchi, S. Matsugo, Four chemotypes of the terrestrial cyanobacterium Nostoc commune characterized by differences in the mycosporine-like amino acids, Phycol. Res. 67 (2019) 3–11.

Publisher's Note: MDPI stays neutral with regard to jurisdictional claims in published maps and institutional affiliations.

© 2020 by the authors. Submitted for possible open access publication under the terms and conditions of the Creative Commons Attribution (CC BY) license (http://creativecommons.org/licenses/by/4.0/).
